# Synthesis of Hydrazidoureidobenzensulfonamides Incorporating a Nicotinoyl Tail and Their Carbonic Anhydrase I, II, IX and XII Inhibitory Activity

**DOI:** 10.3390/ph19020290

**Published:** 2026-02-09

**Authors:** Alberto Deplano, Davide Moi, Serena Vittorio, Andrea Angeli, Claudiu T. Supuran, Valentina Onnis

**Affiliations:** 1 Dipartimento di Scienze della Vita e dell’Ambiente, Cittadella Universitaria di Monserrato, Università degli Studi di Cagliari, S.P. 8 CA, 09042 Monserrato, Italy; alberto.deplano@unica.it (A.D.); davide.moi@unica.it (D.M.); 2 Dipartimento di Scienze Farmaceutiche, Università degli Studi di Milano, Via Mangiagalli 25, 20133 Milano, Italy; 3 Laboratorio di Chimica Bioinorganica, Polo Scientifico Neurofarba Department, Università degli Studi di Firenze, Room 188, Via della Lastruccia 3, Sesto Fiorentino, 50019 Florence, Italy; andrea.angeli@unifi.it (A.A.);

**Keywords:** carbonic anhydrase enzyme inhibition, nicotinoylureas, sulfonamides

## Abstract

**Background**: Carbonic anhydrases (CAs) are known to play important roles in several physiological and pathological processes; among them, CAs IX and XII are of particular relevance in cancer therapy due to their involvement in tumor growth and progression. **Methods**: In this study, a novel series of benzenesulfonamides incorporating a hydrazinocarbonyl-ureido linker alongside a 6-arylpyridine tail was synthesized and evaluated for inhibitory activity through a stopped-flow CO_2_ hydrase assay on four hCA isoforms. **Results**: Some of the new compounds exhibited great activity and selectivity toward the tumor-expressed CA XII isoform over the off-target isoforms CA I and CA II. Based on these results, they were selected for ADME prediction studies, showing favorable drug-like properties. To further investigate their binding mode, these compounds were docked into the four hCA isoforms. **Conclusions**: Overall, the results underscore the potential of compounds bearing a 6-arylpyridine tail along with a hydrazinocarbonyl-ureido linker as a foundation for further inhibitor development.

## 1. Introduction

**Figure 1:**
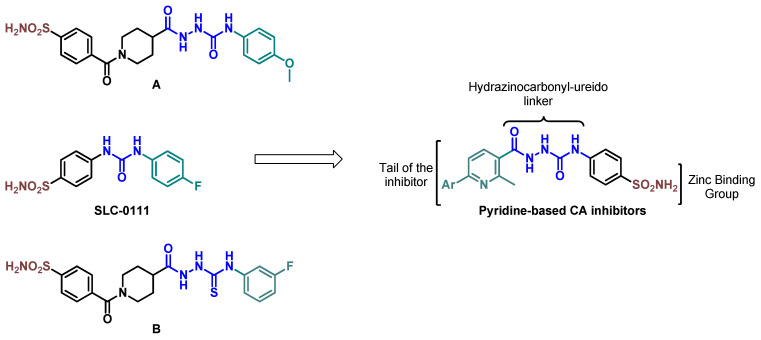
Rational design of the new class of pyridine-based carbonic anhydrase inhibitors.

Carbonic anhydrases (CAs) are ubiquitous metallo-enzymes which catalyze the reversible hydration of carbon dioxide to a bicarbonate ion and a proton [1] (CO_2_ + H_2_O ⇆ HCO_3_^−^ + H^+^). CAs are encoded by eight different genetic families: α-, β-, γ-, δ-, ζ-, η-, and θ-; human CAs (hCAs) belong to the α-class, which features a zinc ion as a cofactor. To date, at least 15 different isoforms have been described, differing in sequence, biochemical properties, distribution in organs and tissues, kinetic properties, and subcellular localization [2]. Of these isoforms, only twelve were found to be catalytically active (I, II, III, IV, VA, VB, VI, VII, IX, XII, XIII, and XIV), while three isoforms, known as CA-related proteins (CARPs VIII, X, and XI), lack any catalytic activity [3]. Apart from differing in the previously mentioned characteristics, these isoforms feature a different domain organization: cytosolic (CAs I, II, III, VII and XIII and CARPs VIII, X and XI), mitochondrial (CAs VA and VB), and secreted (CA VI) isoforms consist solely of the catalytic domain, while transmembrane isoforms (CAs IX, XII and XIV) also present a transmembrane region and an intracellular tail; in addition to this, CA IX also features an extracellular portion, known as a proteoglycan-like domain [4]. Several studies have demonstrated the importance of CAs in several physiological and pathological processes including glaucoma, obesity, osteoporosis, cancer, high-altitude sickness, epilepsy, neuropathic pain, and sleep apnea [5]. Although numerous efforts have been made, the development of selective inhibitors is still ongoing. No inhibitors currently in clinical use are selective for a single isoform, limiting their usefulness due to off-target and adverse effects [6]. The hCA transmembrane isoforms IX and XII are extensively studied as onco-targets due to their overexpression and role in solid tumors [7]. Under healthy conditions, CA IX is mainly expressed on the basolateral surface of epithelial cells, in the gallbladder and in the small intestine, while it is overexpressed in several tumor tissues, and its overexpression is correlated with poor prognosis [8]. This isoform’s expression is mainly regulated by the hypoxia-inducible factor (HIF-1alpha), which explains its high expression under hypoxic conditions [9]. On the other hand, CA XII is more widespread in healthy tissues, and it is also overexpressed in tumors when compared to normal cells [10]. In contrast to CA IX, CA XII expression is not mainly regulated by HIF-1alpha, but it was demonstrated that the von Hippel–Lindau (VHL) tumor suppressor protein can control the expression of the CA XII gene; in fact, it was found that CA is up-regulated in VHL-defective cells [11]. Both isoforms play a pivotal role in tumor proliferation, acidification and progression, regulating both intracellular and extracellular pH, also proving the role of CA XII in drug resistance [12,13,14]. The most common strategy used for the inhibition of hCAs is through molecules that can coordinate the zinc ion in the active site, in particular, sulfonamides and their isosters, carboxylates, dithiocarbamates or hydroxamates [15,16]. Even though inhibitors containing a sulfonamide group are known to be highly potent and have been widely used for the treatment of different pathological conditions, they show poor isoform selectivity, leading to the development of side effects [16,17,18,19]. To increase isoform-specific selectivity, the “tail approach” has emerged as the most efficient method and has been widely adopted. Molecules developed with this approach contain a zinc binding group, a linker and one or more tails; the latter could increase selectivity by binding to the hydrophobic or hydrophilic regions of hCA active sites [20,21]. The efficacy of this method was well validated by the development of the lead compound SLC-0111. This molecule, characterized by a benzensulfonamide moiety as a zinc binder, a urea linker and a 4-fluorophenyl group as a tail, is known to be selective for the isoforms IX and XII. This compound demonstrated its safety during phase I and phase II clinical trials; it also showed antitumor activity alone and in combination with other anticancer agents in preclinical studies [22,23,24]. The main feature of this lead compound is the presence of the ureido linker [25]. It was demonstrated in previous work by our group that the ureido group is able to give high CA selectivity to sulfonamide derivatives; this is due to the high flexibility conferred by this linker, allowing inhibitors to adopt suitable conformations when binding to the enzyme [26]. Moreover, compounds containing ureido and thioureido moieties have shown selective inhibitory properties against different CA isoforms [25,26,27,28]. In previous studies by our group, we also demonstrated the enhanced activity and selectivity of compounds containing hydrazidoureido or hydrazidothioureido linkers [29,30], showing the great potential for CA inhibition of compounds featuring these moieties. On the other hand, pyridine is a privileged ring system in medicinal chemistry due to its wide range of biological activities and well-established medicinal and pharmacological properties, making pyridine derivatives valuable in the treatment of various diseases [31,32]. Recently, a study showed the great activity of structural analogues of SLC-0111, where the 4-fluorophenyl tail was substituted by a 6-arylpyridine moiety. These compounds have shown favorable activity and selectivity, with no noticeable toxicity toward healthy cells [33]. As a continuation of our previous studies in this field, here we developed a small library of benzenesulfonamides linked to 6-arylpyridine derivatives, using a hydrazidoureido linker (Figure 1). These new compounds were then tested against hCA I and hCA II as off-target isoforms and hCAs IX and XII as target isoforms. To better understand the binding interactions with the various isoforms and to corroborate the inhibition data, docking studies were also performed.

## 2. Results and Discussion

### 2.1. Chemistry

**Scheme 1:**
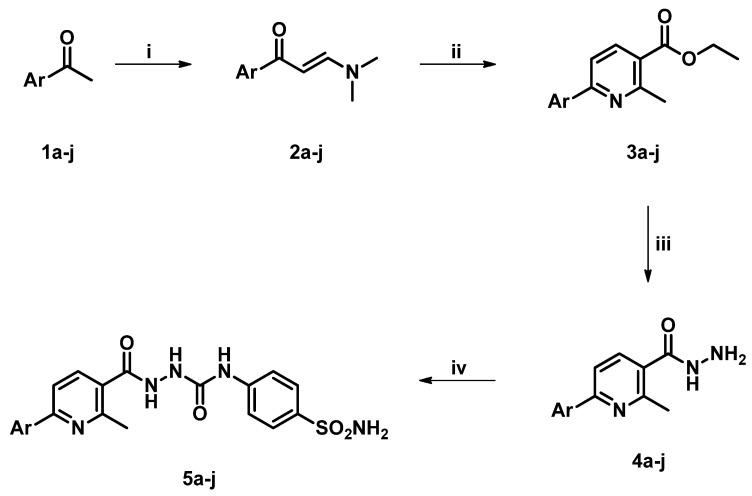
Synthetic procedure for hydrazidoureidosulfonamides **5a-j**. Reagents and conditions: (i) DMF-DMA, and reflux for 12 h; (ii) ethyl acetoacetate, ammonium acetate, acetic acid, and reflux for 2 h; (iii) hydrazine hydrate, ethanol, and reflux for 5 h; and (iv) phenyl(4-sulfamoylphenyl)carbamate, DMF, and stirring 24 h.

The desired compounds were obtained through the following synthetic pathway. The intermediate enaminone derivatives **2** were prepared by reaction of aryl ketones **1** with dimethylformamide dimethyl acetal (DMF-DMA) at reflux. The resulting enaminones **2** were heterocyclized to ethyl-2-methyl-6-arylnicotinates **3**, by refluxing with ethyl acetoacetate and sodium acetate in an acetic acid solution. The obtained compounds **3** were then treated with hydrazine hydrate in absolute ethanol to afford the hydrazide derivatives **4**. The final step was the reaction between the hydrazides **4** and phenyl(4-sulfamoylphenyl)carbamate in DMF to obtain the hydrazidoureido derivatives **5**. The chemical structure of the synthesized compounds was confirmed by ^1^H NMR, ^13^C NMR, and IR spectroscopy (see Supplementary Materials), which showed the characteristic signals for aromatic and NH moieties, as well as by elemental analyses, whose results were fully consistent with the proposed structures (Scheme 1).

### 2.2. Carbonic Anhydrase Inhibition Assays

The novel sulfonamide derivatives **5a-j** were tested for their enzymatic inhibitory activity against the four isoforms of hCA, the two off-target isoforms hCA I and II and the two cancer-related isoforms hCA IX and XII, by a stopped-flow CO_2_ hydrase assay using the standard inhibitor acetazolamide as a positive control (Table 1) [34].

The sulfonamide derivatives **5** exhibited activity toward cytosolic isoform hCA I, with inhibition values spanning from 21.4 nM to 750.4 nM. Compound **5d**, which bears a bromine atom at the 3-position of the aryl ring, exhibited the highest activity toward this isoform. Substitution of the bromine with a methyl group (compound **5c**) resulted in an activity reduction. We also explored the effects of different substituents in other positions of the aryl ring; in particular, compound **5e** possessing a fluorine at the 4-position showed an inhibition value of 32.5 nM. The replacement of the fluorine with trifluoromethyl (compound **5f**) produced a reduction in activity. Moreover, substitution with a methansulfonamide (compound **5i**) or an acetamide group (compound **5j**) resulted in a drastic reduction in the activity by about 23-fold and 3-fold respectively. The presence of a 3,4,5-trimethoxyphenyl group (compound **5g**) resulted in greater activity compared to a 3,4-dimethoxyphenyl analogue (compound **5h**). The presence of 3,5-bis(trifluoromethyl)phenyl (compound **5a**) drastically reduced the activity to an inhibition value of 298.2 nM. We also tested the effects of the substitution of the aryl ring with a benzofuran (compound **5b**), which showed an inhibition value of 77.5 nM.

The dominant cytosolic isoform hCA II was effectively inhibited by most of the synthesized compounds with inhibition values ranging between 3.8 nM and 223.0 nM. Compounds **5a**-**5e** and **5h** showed the greatest activity. Compound **5h** showed the best activity, while a drastic reduction in activity of about 9-fold was observed when a supplementary methoxy group was introduced at the 5-position (compound **5g**). Substitution of the aryl ring at the 4-position with an acetamide (compound **5j**), a methansulfonamide (compound **5i**) or a trifluoromethyl group (compound **5f**) resulted in a decrease in inhibitory activity, with compound **5f** being the weakest hCA II inhibitor.

Regarding the transmembrane isoform hCA IX, the best activity was observed with compound **5d**, which features a 3-bromophenyl ring, with an inhibition value of 22.7 nM. The substitution of the bromine in the same position with a methyl group (compound **5c**) drastically reduced the activity by about 6-fold. Other substitutions at other positions also resulted in an activity reduction, with compound **5i** being the least active compound. Substitution of the 4-methansulfonamide group of **5i** with an acetamide, fluorine or trifluoromethyl group to give, respectively, compound **5j**, compound **5e** or compound **5f** resulted in a partial recovery of activity. Comparable activity was also observed for the 3,4,5-trimethoxyphenyl derivative (compound **5g**) and for the benzofuran derivative (compound **5b**). Substitution with a 3,5-bis(trifluoromethyl)phenyl group (compound **5a**) and a 3,4-(dimethoxyphenyl) group (compound **5h**) also resulted in a great loss of activity toward the isoform hCA IX.

On the other hand, all compounds showed a good inhibition profile toward the isoform hCA XII, showing inhibition values ranging from 5.8 nM to 56.2 nM; compound **5f**, compound **5i** and compound **5j** also demonstrated good selectivity toward this isoform. Compound **5f,** bearing a 4-fluorophenyl moiety, was the most active, with an inhibition value of 5.8 nM, a CA II/CA XII selectivity index of 8 and a CA I/CA XII selectivity index of 13. The replacement of 4-fluorine with 4-methansulfonamide (compound **5i**) produced a CA I/CA XII selectivity index of 96, while replacement with 4-acetamide (compound **5j**) reduced the CA I/CA XII selectivity index to 10. The compounds that showed the lowest activity were the benzofuran derivative **5b** and 3-bromophenyl derivative **5d**; these compounds showed inhibition values of 47.7 nM and 56.2 nM, respectively, and also greater selectivity toward the cytosolic isoform hCA II over the transmembrane, tumor-associated isoform hCA XII, with CA II/CA XII selectivity index values of 0.20 and 0.10, respectively.

### 2.3. ADME Predictions

Compounds **5f**, **5i** and **5j**, endowed with both potency and selectivity against hCA XII, were further selected to evaluate their predicted drug-like properties through the SwissADME (Absorption, Distribution, Metabolism, Excretion) online tool [35], showing favorable pharmacokinetic properties, as shown in Table 2. Different crucial parameters were predicted, such as the predicted solubility, the LogP and the number of H-bond acceptors and donors. Moreover, it was also calculated if the selected compounds could be substrates or non-substrates of the permeability glycoprotein P-gp, in combination with BBB permeation, which can be useful to verify the brain permeability of the selected molecules. Concerning physicochemical properties, the selected compounds displayed acceptable solubility in water, according to the ESOL solubility parameter, which may favor drug formulation. The consensus Log-P values were calculated to be between 0.86 and 2.66; none of these compounds were found to be substrates for P-glycoproteins. Furthermore, the tested compounds showed no blood−brain barrier penetration and a bioavailability score between 0.55 and 0.17.

### 2.4. Docking Studies

**Figure 2:**
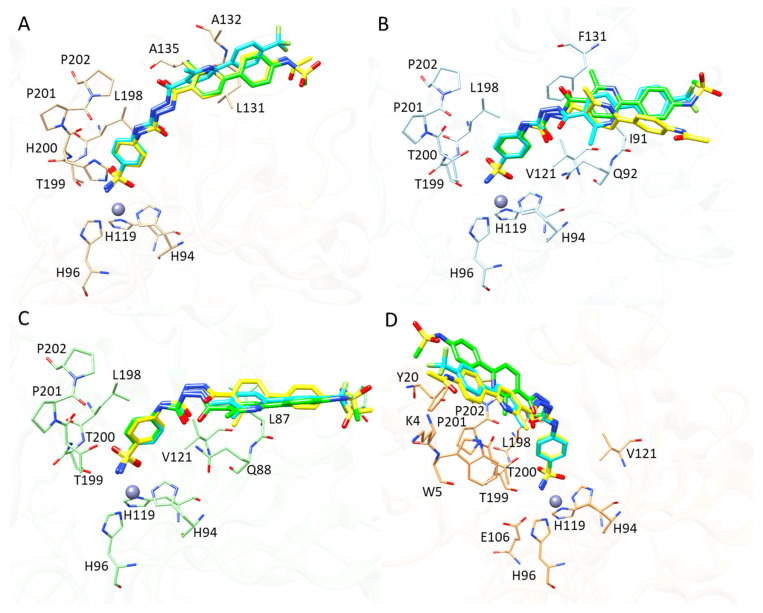
Docking poses of compounds **5f** (cyan sticks), **5i** (green sticks), and **5j** (yellow sticks) within hCA I—Panel (**A**); hCA II—Panel (**B**); hCA IX—Panel (**C**); and hCA XII—Panel (**D**).

Molecular docking studies were conducted to elucidate the binding modes of inhibitors **5f**, **5i**, and **5j**, which exhibited higher inhibitory activity toward the tumor-associated isoform hCA XII, to the studied hCA isoforms. As depicted in Figure 2, all analyzed compounds bind within the catalytic pocket of hCAs, with the benzensulfonamide moiety coordinating the Zn^2+^ ion and being further stabilized by a H-bond with T199 and hydrophobic contacts with L198, in agreement with the typical binding mode adopted by this class of hCAIs. In addition, this moiety is also implicated in hydrophobic interactions with V121 in all investigated hCA isoforms, except for hCA I.

Within the hCA I active site, the arylpyridine system is involved in hydrophobic contacts with A132, A135 and L131 (Figure 2A). In hCA II, the pyridine ring of the inhibitors elicits hydrophobic contacts with I91, while the carbonyl group of compounds **5i** and **5j** establishes a H-bond with Q92, which might account for their greater affinity toward hCA II compared to **5f** (Figure 2B). Furthermore, the methyl group of **5i** engages in hydrophobic interactions with F131. Regarding hCA IX, all analyzed compounds form a H-bond between their carbonyl group and Q88, along with hydrophobic interactions between the pyridine ring and L87 (Figure 2C). Interestingly, within the hCA XII binding site, the tails of **5f**, **5i**, and **5j** assume a markedly different orientation with respect to the other investigated hCA isoforms, which may underlie their enhanced selectivity for hCA XII (Figure 2D). In more detail, the arylpyridine portion of **5f**, **5i**, and **5j** establishes hydrophobic contacts with P201, P202 and K4, as well as pi-stacking interactions with W5 and Y20. In addition, the sulfonamide moiety is also implicated in a H-bond with E106, which may further contribute to the increased affinity of these compounds for hCA XII.

## 3. Materials and Methods

### 3.1. Chemistry

All commercially available solvents and reagents were used without further purification. ^1^H NMR spectra for compounds **3a-j**, **4a-j** and **5a-j** were recorded on a Bruker Avance III HD 600 spectrometer (Bruker, Bremen, Germany). The chemical shifts (δ) are reported in parts per million downfield from tetramethylsilane (TMS), which was used as the internal standard. The spectra were recorded in hexadeuteriodimethylsulfoxide (DMSO-*d*_6_). Infrared spectra were recorded on a Nicolet iS10 spectrometer (Thermo Fisher Scientific Inc., Paisley, UK). The main bands are given in cm^−1^. Positive-ion electrospray ionization (ESI) mass spectra were recorded on a double-focusing MAT 95 instrument (Finnigan, Waltham, MA, USA) with BE geometry. Melting points (mps) were determined with an SMP1 Melting Point apparatus (Stuart Scientific, Stone, UK) and are uncorrected. All products reported showed ^1^H NMR spectra in agreement with the assigned structures. Compounds **2a-j** were prepared as previously described [36,37,38,39,40,41,42,43]. The purity of the tested compounds was determined by combustion elemental analyses conducted by the Microanalytical Laboratory of the Chemistry Department of the University of Ferrara with an MT-5 CHN recorder elemental analyzer (Yanagimoto, Kyoto, Japan), and the values found were within 0.4% of theoretical values.

#### 3.1.1. General Procedure for the Preparation of Ethyl 2-methyl-6-phenylnicotinates (**3a-j**)

(*E*)-3-(Dimethylamino)-1-phenylprop-2-en-1-ones (5 mmol) **2a-j** were dissolved in glacial acetic acid (10 mL); then, ammonium acetate (3.08 g, 40 mmol) was added, followed by ethyl acetoacetate (0.76 mL, 6 mmol). The reaction mixture was refluxed for 12 h and then cooled down to room temperature. The reaction mixture was poured into water at 0 °C, and the formed solids were filtered off and washed with water, giving the corresponding ethyl nicotinates **3a-j**.

##### Ethyl 6-(3,5-bis(trifluoromethyl)phenyl)-2-methylnicotinate (**3a**)

Following the general procedure, the title compound was prepared starting from **2a**. Yield 77%; M.p. 147–148 °C. ^1^H NMR (DMSO-*d*_6_) δ 8.77 (s, 2H, Ar), 8.30 (d, *J* = 8.2 Hz, 1H, Ar), 8.26 (d, *J* = 8.2 Hz, 1H, Ar), 8.22 (s, 1H, Ar), 4.35 (q, *J* = 7.1 Hz, 2H, CH_2_), 2.82 (s, 3H, CH_3_), 1.36 (t, *J* = 7.1 Hz, 3H, CH_3_). IR 3022, 1722, 1684 cm^−1^. Elemental analysis calculated for C_17_H_13_F_6_NO_2_ (377.28): %C, 54.12; %H, 3.47; %N, 3.71. Found: %C, 54.11; %H, 3.47; %N, 3.73. *m*/*z* 378.

##### Ethyl 6-(benzofuran-2-yl)-2-methylnicotinate (**3b**)

Following the general procedure, the title compound was prepared starting from **2b**. Yield 61%; M.p. 162–163 °C. ^1^H NMR (DMSO-*d*_6_) δ 8.31 (d, *J* = 8.3 Hz, 1H, Ar), 7.90 (d, *J* = 8.2 Hz, 1H, Ar), 7.76 (d, *J* = 7.9 Hz, 1H, Ar), 7.68–7.72 (m, 2H, Ar), 7.42 (t, *J* = 7.8 Hz, 1H, Ar), 7.32 (t, *J* = 7.5 Hz, 1H, Ar), 4.34 (q, *J* = 7.1 Hz, 2H, CH_2_), 2.80 (s, 3H, CH_3_), 1.35 (t, *J* = 7.1 Hz, 3H, CH_3_). IR 3009, 1728, 1677 cm^−1^. Elemental analysis calculated for C_17_H_15_NO_3_ (281.31): %C, 72.58; %H, 5.37; %N, 4.98. Found: %C, 72.57; %H, 5.38; %N, 4.99. *m*/*z* 282.

##### Ethyl 2-methyl-6-(m-tolyl)nicotinate (**3c**)

Following the general procedure, the title compound was prepared starting from **2c**. Yield 82%; M.p. 155–157 °C. ^1^H NMR (DMSO-*d*_6_) δ 8.21 (d, *J* = 8.2 Hz, 1H, Ar), 7.96 (s, 1H, Ar), 7.92 (d, *J* = 7.8 Hz, 1H, Ar), 7.88 (d, *J* = 8.3 Hz, 1H, Ar), 7.39 (t, *J* = 7.7 Hz, 1H, Ar), 7.29 (d, *J* = 7.5 Hz, 1H, Ar), 4.32 (q, *J* = 7.2 Hz, 2H, CH_2_), 2.78 (s, 3H, CH_3_), 2.39 (s, 3H, CH_3_), 1.34 (t, *J* = 7.2 Hz, 3H, CH_3_). IR 3102, 1736, 1692 cm^−1^. Elemental analysis calculated for C_16_H_17_NO_2_ (255.31): %C, 75.27; %H, 6.71; %N, 5.49. Found: %C, 75.26; %H, 6.72; %N, 5.49. *m*/*z* 256.

##### Ethyl 6-(3-bromophenyl)-2-methylnicotinate (**3d**)

Following the general procedure, the title compound was prepared starting from **2d**. Yield 49%; M.p. 142–143 °C. ^1^H NMR (DMSO-*d*_6_) δ 8.33 (s, 1H, Ar), 8.24 (d, *J* = 8.3 Hz, 1H, Ar), 8.14 (d, *J* = 7.9 Hz, 1H, Ar), 7.98 (d, *J* = 8.3 Hz, 1H, Ar), 7.68 (d, *J* = 7.9 Hz, 1H, Ar), 7.48 (t, *J* = 7.9 Hz, 1H, Ar), 4.33 (q, *J* = 7.2 Hz, 2H, CH_2_), 2.79 (s, 3H, CH_3_), 1.34 (t, *J* = 7.2 Hz, 3H, CH_3_). IR 3112, 1729, 1699 cm^−1^. Elemental analysis calculated for C_15_H_14_BrNO_2_ (320.18): %C, 56.27; %H, 4.41; %N, 4.37. Found: %C, 56.29; %H, 4.40; %N, 4.39. *m*/*z* 321.

##### Ethyl 6-(4-fluorophenyl)-2-methylnicotinate (**3e**)

Following the general procedure, the title compound was prepared starting from **2e**. Yield 65%; M.p. 139–140 °C. ^1^H NMR (DMSO-*d*_6_) δ 8.18–8.25 (m, 3H, Ar), 7.92 (d, *J* = 8.3 Hz, 1H, Ar), 7.34 (t, *J* = 8.8 Hz, 2H, Ar), 4.33 (q, *J* = 7.1 Hz, 2H, CH_2_), 2.78 (s, 3H, CH_3_), 1.34 (t, *J* = 7.1 Hz, 3H, CH_3_). IR 3122, 1736, 1684 cm^−1^. Elemental analysis calculated for C_15_H_14_FNO_2_ (259.28): %C, 69.49; %H, 5.44; %N, 5.40. Found: %C, 69.49; %H, 5.42; %N, 5.39. *m*/*z* 260.

##### Ethyl 2-methyl-6-(4-(trifluoromethyl)phenyl)nicotinate (**3f**)

Following the general procedure, the title compound was prepared starting from **2f**. Yield 59%; M.p. 155–156 °C. ^1^H NMR (DMSO-*d*_6_) δ 8.33 (d, *J* = 8.5 Hz, 2H, Ar), 8.27 (d, *J* = 8.4 Hz, 1H, Ar), 8.01 (d, *J =* 8.4 Hz, 1H, Ar), 7.85 (d, *J* = 8.3, 2H, Ar); 4.33 (q, *J* = 7.2 Hz, 2H, CH_2_), 2.79 (s, 3H, CH_3_), 1.34 (t, *J* = 7.0 Hz, 3H, CH_3_). IR 3110, 1741, 1698 cm^−1^. Elemental analysis calculated for C_16_H_14_F_3_NO_2_ (309.28): %C, 62.13; %H, 4.56; %N, 4.53. Found: %C, 62.15; %H, 4.57; %N, 4.51. *m*/*z* 310.

##### Ethyl 2-methyl-6-(3,4,5-trimethoxyphenyl)nicotinate (**3g**)

Following the general procedure, the title compound was prepared starting from **2g**. Yield 41%; M.p. 147–149 °C. ^1^H NMR (DMSO-*d*_6_) δ 8.21 (d, *J* = 8.3 Hz, 1H, Ar), 7.97 (d, *J* = 8.3 Hz, 1H, Ar), 7.46 (s, 2H, Ar), 4.33 (q, *J* = 7.1 Hz, 2H, Ar), 3.89 (s, 6H, CH_3_), 3.73 (s, 3H, CH_3_), 2.79 (s, 3H, CH_3_), 1.34 (t, *J* = 7.1 Hz, 3H, CH_3_). IR 3119, 1728, 1679 cm^−1^. Elemental analysis calculated for C_18_H_21_NO_5_ (331.36): %C, 65.24; %H, 6.39; %N, 4.23. Found: %C, 65.22; %H, 6.41; %N, 4.22. *m*/*z* 332.

##### Ethyl 2-methyl-6-(3,4-dimethoxyphenyl)nicotinate (**3h**)

Following the general procedure, the title compound was prepared starting from **2h**. Yield 41%; M.p. 152–153 °C. ^1^H NMR (DMSO-*d*_6_) δ 8.19 (d, *J* = 8.3 Hz, 1H, Ar), 7.89 (d, *J* = 8.3 Hz, 1H, Ar), 7.72–7.76 (m, 2H, Ar), 7.07 (d, *J* = 8.4 Hz, 1H, Ar), 4.32 (q, *J* = 7.1 Hz, 2H, CH_2_), 3.86 (s, 3H, CH_3_), 3.83 (s, 3H, CH_3_), 2.78 (s, 3H, CH_3_), 1.34 (t, *J* = 7.1 Hz, 3H, CH_3_). IR 3121, 1726, 1674 cm^−1^. Elemental analysis calculated for C_17_H_19_NO_4_ (301.34): %C, 67.76; %H, 6.36; %N, 4.65. Found: %C, 67.75; %H, 6.36; %N, 4.66. *m*/*z* 302.

##### Ethyl 2-methyl-6-(4-(methylsulfonamido)phenyl)nicotinate (**3i**)

Following the general procedure, the title compound was prepared starting from **2i**. Yield 63%; M.p. 166–168 °C. ^1^H NMR (DMSO-*d*_6_) δ 10.11 (s, 1H, NH), 8.31 (s, 1H, Ar), 8.26 (d, *J* = 8.3 Hz, 1H, Ar), 7.84 (d, *J* = 8.2 Hz, 1H, Ar), 7.75–7.81 (m, 2H, Ar), 7.43 (t, *J* = 7.9 Hz, 1H, Ar), 4.33 (q, *J* = 7.2 Hz, 2H, CH_2_), 2.80 (s, 3H, CH_3_), 2.07 (s, 3H, CH_3_), 1.34 (t, *J* = 7.1 Hz, 3H, CH_3_). IR 3223, 1733, 1682 cm^−1^. Elemental analysis calculated for C_16_H_18_N_2_O_4_S (334.40): %C, 57.47; %H, 5.43; %N, 8.38. Found: %C, 57.45; %H, 5.44; %N, 8.37. *m*/*z* 335.

##### Ethyl 6-(4-acetamidophenyl)-2-methylnicotinate (**3j**)

Following the general procedure, the title compound was prepared starting from **2j**. Yield 77%; M.p. 184–185 °C. ^1^H NMR (DMSO-*d*_6_) δ 8.25 (d, *J* = 8.3 Hz, 1H, NH), 8.17 (d, *J* = 8.7 Hz, 2H, Ar), 7.94 (d, *J* = 8.3 Hz, 1H, Ar), 7.55 (d, *J* = 8.8 Hz, 2H, Ar), 4.33 (q, *J* = 7.1 Hz, 2H, CH_2_), 2.99 (s, 3H, CH_3_), 2.79 (s, 3H, CH_3_), 1.34 (t, *J* = 7.1 Hz, 3H, CH_3_). IR 3237, 1741, 1695 cm^−1^. Elemental analysis calculated for C_17_H_18_N_2_O_3_ (298.34): %C, 68.44; %H, 6.08; %N, 9.39. Found: %C, 68.43; %H, 6.08; %N, 9.38. *m*/*z* 299.

#### 3.1.2. General Procedure for the Preparation of 2-methyl-6-phenylnicotinohydrazides (**4a-j**)

A mixture of Ethyl 2-methyl-6-phenylnicotinates (3 mmol) (**3a-j**) and hydrazine monohydrate (0.5 mL, 10 mmol) in ethanol (EtOH) (20 mL) was refluxed overnight. After cooling, the precipitate formed was filtered off, washed with water and dried, giving the corresponding phenylnicotinohydrazides **4a-j,** which were used in the next step without further purification.

##### 6-(3,5-Bis(trifluoromethyl)phenyl)-2-methylnicotinohydrazide (**4a**)

Following the general procedure, the title compound was prepared starting from **3a**. Yield 84%; M.p. 203–204 °C. ^1^H NMR (DMSO-*d*_6_) δ 9.68 (s, 1H, NH), 8.76 (s, 2H, Ar), 8.20 (d, *J* = 8.0 Hz, 2H, Ar), 7.86 (d, *J* = 8.0 Hz, 1H, Ar), 4.68 (s, 2H, NH_2_), 2.64 (s, 3H, CH_3_). IR 3344, 3022, 1679 cm^−1^. Elemental analysis calculated for C_15_H_11_F_6_N_3_O (363.26): %C, 49.60; %H, 3.05; %N, 11.57. Found: %C, 49.61; %H, 3.07; %N, 11.58. *m*/*z* 364.

##### 6-(Benzofuran-2-yl)-2-methylnicotinohydrazide (**4b**)

Following the general procedure, the title compound was prepared starting from **3b**. Yield 88%; M.p. 222–223 °C. ^1^H NMR (DMSO-*d*_6_) δ 9.64 (s, 1H, NH), 7.83 (s, 2H, Ar), 7.74 (d, *J* = 7.6 Hz, 1H, Ar), 7.68 (d, *J* = 8.3 Hz, 1H, Ar), 7.61 (s, 1H, Ar), 7.38–7.42 (m, 1H, Ar), 7.31 (t, *J* = 7.5 Hz, 1H, Ar), 4.54 (s, 2H, NH_2_), 2.61 (s, 3H, CH_3_). IR 3329, 3108, 1682 cm^−1^. Elemental analysis calculated for C_15_H_13_N_3_O_2_ (267.28): %C, 67.40; %H, 4.90; %N, 15.72. Found: %C, 67.40; %H, 4.88; %N, 15.73. *m*/*z* 268.

##### 2-Methyl-6-(m-tolyl)nicotinohydrazide (**4c**)

Following the general procedure, the title compound was prepared starting from **3c**. Yield 74%; M.p. 201–202 °C. ^1^H NMR (DMSO-*d*_6_) δ 9.59 (s, 1H, NH), 7.93 (s, 1H, Ar), 7.88 (d, *J* = 8.0 Hz, 1H, Ar), 7.81 (d, *J* = 8.0 Hz, 1H, Ar), 7.75 (d, *J* = 8.0 Hz, 1H, Ar), 7.38 (t, *J* = 7.6 Hz, 1H, Ar), 7.26 (d, *J* = 8.3 Hz, 1H, Ar), 4.52 (s, 2H, NH_2_), 2.60 (s, 3H, CH_3_), 2.40 (s, 3H, CH_3_). IR 3342, 3115, 1674 cm^−1^. Elemental analysis calculated for C_14_H_15_N_3_O (241.29): %C, 69.69; %H, 6.27; %N, 17.41. Found: %C, 69.67; %H, 6.28; %N, 17.40. *m*/*z* 242.

##### 6-(3-Bromophenyl)-2-methylnicotinohydrazide (**4d**)

Following the general procedure, the title compound was prepared starting from **3d**. Yield 80%; M.p. 223–225 °C. ^1^H NMR (DMSO-*d*_6_) δ 9.62 (s, 1H, NH), 8.30 (s, 1H, Ar), 8.11 (d, *J* = 8.0 Hz, 1H, Ar), 7.90 (d, *J* = 8.0 Hz, 1H, Ar), 7.78 (d, *J* = 8.0 Hz, 1H, Ar), 7.65 (d, *J* = 7.9 Hz, 1H, Ar), 7.47 (t, *J* = 7.9 Hz, 1H, Ar), 4.54 (s, 2H, NH_2_), 2.60 (s, 3H, CH_3_). IR 3324, 3108, 1669 cm^−1^. Elemental analysis calculated for C_13_H_12_BrN_3_O (306.16): %C, 51.00; %H, 3.95; %N, 13.72. Found: %C, 51.01; %H, 3.96; %N, 13.72. *m*/*z* 307.

##### 6-(4-Fluorophenyl)-2-methylnicotinohydrazide (**4e**)

Following the general procedure, the title compound was prepared starting from **3e**. Yield 78%; M.p. 220–222 °C. ^1^H NMR (DMSO-*d*_6_) δ 9.59 (s, 1H, NH), 8.14–8.18 (m, 2H, Ar), 7.83 (d, *J* = 8.0 Hz, 1H, Ar), 7.76 (d, *J* = 8.0 Hz, 1H, Ar), 7.32 (t, *J* = 8.9 Hz, 2H, Ar), 4.52 (s, 2H, NH_2_), 2.60 (s, 3H, CH_3_). IR 3343, 3119, 1672 cm^−1^. Elemental analysis calculated for C_13_H_12_FN_3_O (245.25): %C, 63.66; %H, 4.93; %N, 17.13. Found: %C, 63.66; %H, 4.95; %N, 17.12. *m*/*z* 246.

##### 2-Methyl-6-(4-(trifluoromethyl)phenyl)nicotinohydrazide (**4f**)

Following the general procedure, the title compound was prepared starting from **3f**. Yield 69%; M.p. 208–210 °C. ^1^H NMR (DMSO-*d*_6_) δ 9.64 (s, 1H, NH), 8.32 (d, *J* = 8.3 Hz, 2H, Ar), 7.95 (d, *J* = 8.0 Hz, 1H, Ar), 7.86 (d, *J* = 8.4 Hz, 2H, Ar), 7.83 (d, *J* = 8.0 Hz, 1H, Ar), 4.55 (s, 2H, NH_2_), 2.62 (s, 3H, CH_3_). IR 3339, 3124, 1683 cm^−1^. Elemental analysis calculated for C_14_H_12_F_3_N_3_O (295.26): %C, 56.95; %H, 4.10; %N, 14.23. Found: %C, 56.96; %H, 4.08; %N, 14.22. *m*/*z* 246.

##### 2-Methyl-6-(3,4,5-trimethoxyphenyl)nicotinohydrazide (**4g**)

Following the general procedure, the title compound was prepared starting from **3g**. Yield 89%; M.p. 238–239 °C. ^1^H NMR (DMSO-*d*_6_) δ 9.58 (s, 1H, NH), 7.88 (d, *J* = 7.9 Hz, 1H, Ar), 7.75 (d, *J* = 8.2 Hz, 1H, Ar), 7.41 (s, 2H, Ar), 4.52 (s, 2H, NH_2_), 3.88 (s, 6H, CH_3_), 3.72 (s, 3H, CH_3_), 2.60 (s, 3H, CH_3_). IR 3327, 3109, 1672 cm^−1^. Elemental analysis calculated for C_16_H_19_N_3_O_4_ (317.34): %C, 60.56; %H, 6.03; %N, 13.24. Found: %C, 60.56; %H, 6.02; %N, 13.25. *m*/*z* 318.

##### 6-(3,4-Dimethoxyphenyl)-2-methylnicotinohydrazide (**4h**)

Following the general procedure, the title compound was prepared starting from **3h**. Yield 81%; M.p. 227–228 °C. ^1^H NMR (DMSO-*d*_6_) δ 9.56 (s, 1H, Ar), 7.80 (d, *J* = 8.1 Hz, 1H, Ar), 7.67–7.73 (m, 3H, Ar), 7.06 (d, *J* = 8.3 Hz, 1H, Ar), 4.51 (s, 2H, NH_2_), 3.85 (s, 3H, CH_3_), 3.82 (s, 3H, CH_3_), 2.59 (s, 3H, CH_3_). IR 3319, 3115, 1673 cm^−1^. Elemental analysis calculated for C_15_H_17_N_3_O_3_ (387.32): %C, 62.71; %H, 5.96; %N, 14.63. Found: %C, 62.72; %H, 5.97; %N, 14.63. *m*/*z* 388.

##### N-(4-(5-(Hydrazinecarbonyl)-6-methylpyridin-2-yl)phenyl)methanesulfonamide (**4i**)

Following the general procedure, the title compound was prepared starting from **3i**. Yield 76%; M.p. 244–246 °C. ^1^H NMR (DMSO-*d*_6_) δ 10.09 (s, 1H, NH), 9.59 (s, 1H, Ar), 8.25 (s, 1H, Ar), 7.70–7.79 (m, 4H, Ar), 7.41 (t, *J* = 7.9 Hz, 1H, Ar), 4.50 (s, 2H, NH_2_), 2.60 (s, 3H, CH_3_), 2.07 (s, 3H, CH_3_). IR 3317, 3208, 1662 cm^−1^. Elemental analysis calculated for C_14_H_16_N_4_O_3_S (320.37): %C, 52.49; %H, 5.03; %N, 17.49. Found: %C, 52.50; %H, 5.03; %N, 17.49. *m*/*z* 321.

##### N-(4-(5-(Hydrazinecarbonyl)-6-methylpyridin-2-yl)phenyl)acetamide (**4j**)

Following the general procedure, the title compound was prepared starting from **35**. Yield 64%; M.p. 239–241 °C. ^1^H NMR (DMSO-*d*_6_) δ 9.60 (s, 1H, NH), 8.13 (d, *J* = 8.7 Hz, 2H, Ar), 7.85 (d, *J* = 8.0 Hz, 1H, Ar), 7.77 (d, *J* = 8.0 Hz, 1H, Ar), 7.53 (d, *J* = 8.8 Hz, 2H, Ar), 4.52 (s, 2H, NH_2_), 2.99 (s, 3H, CH_3_), 2.60 (s, 3H, CH_3_). IR 3333, 3217, 1670 cm^−1^. Elemental analysis calculated for C_15_H_16_N_4_O_2_ (284.31): %C, 63.37; %H, 5.67; %N, 19.71. Found: %C, 63.35; %H, 5.68; %N, 19.69. *m*/*z* 285.

#### 3.1.3. General Procedure for the Preparation of 2-(Aryl)-2-methylnicotinoyl)-N-(4-sulfamoylphenyl)hydrazinecarboxamide (**5a-j**)

A mixture of phenyl (4-sulfamoylphenyl)carbamate (0.29 g, 1 mmol) and substituted phenylnicotinohydrazides **4a-j** (1 mmol), in anhydrous DMF (3 mL), was stirred at room temperature for 24 h. Then, water (10 mL) was added, and the mixture was stirred at room temperature until a solid formed. The formed solid was filtered off, washed with water, air-dried and recrystallized from EtOH to give the desired sulfamoylphenyl)hydrazinecarboxamide **5a-j**.

##### 2-(6-(3,5-Bis(trifluoromethyl)phenyl)-2-methylnicotinoyl)-N-(4-sulfamoylphenyl)hydrazinecarboxamide (**5a**)

Following the general procedure, the title compound was prepared starting from **4a**. Yield 43%; M.p. >250 °C. ^1^H NMR (DMSO-*d*_6_) δ 10.31 (s, 1H, NH), 9.33 (s, 1H, NH), 8.79 (s, 2H, Ar), 8.50 (s, 1H, NH), 8.27 (d, *J* = 8.1 Hz, 1H, Ar), 8.22 (s, 1H, Ar), 8.02 (d, *J* = 6.1 Hz, 1H, Ar), 7.73 (d, *J* = 8.8 Hz, 2H, Ar), 7.66 (d, *J* = 8.9 Hz, 2H, Ar), 7.20 (s, 2H, NH_2_), 2.72 (s, 3H, CH_3_). ^13^C NMR (DMSO-*d*_6_) δ 167.5, 162.3, 156.1, 152.6, 142.8, 140.3, 137.1, 137.0, 131.1, 130.8, 130.6, 129.9, 127.1, 124.2, 122.8, 122.4, 120.6, 118.2, 117.7, 35.8, 30.7, 23.0. IR 3348 (stretching -NH), 3262 (stretching -NH), 1662 (stretching -C=O) cm^−1^. Elemental analysis calculated for C_22_H_17_F_6_N_5_O_4_S (482.51): %C, 47.06; %H, 3.05; %N, 12.47. Found: %C, 47.06; %H, 3.03; %N, 12.48. *m*/*z* 483.

##### 2-(6-(Benzofuran-2-yl)-2-methylnicotinoyl)-N-(4-sulfamoylphenyl)hydrazinecarboxamide (**5b**)

Following the general procedure, the title compound was prepared starting from **4b**. Yield 58%; M.p. >250 °C. ^1^H NMR (DMSO-*d*_6_) δ 10.26 (s, 1H, NH), 9.34 (s, 1H, NH), 8.52 (s, 1H, NH), 7.99 (s, 1H, Ar), 7.90 (d, *J* = 8.0 Hz, 1H, Ar), 7.72–7.77 (m, 3H, Ar), 7.70 (d, *J* = 8.3 Hz, 1H, Ar), 7.64–7.69 (m, 3H, Ar), 7.41 (t, *J* = 8.4 Hz, 1H, Ar), 7.30–7.34 (m, 1H, Ar), 7.20 (s, 2H, NH_2_), 2.69 (s, 3H, CH_3_). ^13^C NMR (DMSO-*d*_6_) δ 167.6, 156.6, 154.8, 154.2, 148.4, 142.8, 137.0, 136.8, 129.3 (2C), 129.1, 128.3, 126.7, 125.8, 123.5, 122.0, 117.7, 116.5, 115.2, 111.5, 105.9, 22.9. IR 3322 (stretching -NH), 1662 (stretching -C=O) cm^−1^. Elemental analysis calculated for C_22_H_19_N_5_O_5_S (465.48): %C, 56.77; %H, 4.11; %N, 15.05. Found: %C, 56.76; %H, 4.10; %N, 15.06. *m*/*z* 466.

##### 2-(2-Methyl-6-(m-tolyl)nicotinoyl)-N-(4-sulfamoylphenyl)hydrazinecarboxamide (**5c**)

Following the general procedure, the title compound was prepared starting from **4c**. Yield 76%; M.p. >250 °C. ^1^H NMR (DMSO-*d*_6_) δ 10.20 (s, 1H, NH), 9.30 (s, 1H, NH), 8.46 (s, 1H, NH), 7.96 (s, 1H, Ar), 7.91 (d, *J* = 8.0 Hz, 2H, Ar), 7.88 (d, *J* = 8.1 Hz, 1H, Ar), 7.73 (d, *J* = 8.9 Hz, 2H, Ar), 7.66 (d, *J* = 8.9 Hz, 2H, Ar), 7.40 (t, *J* = 7.7 Hz, 1H, Ar), 7.28 (d, *J* = 7.4 Hz, 1H, Ar), 7.20 (s, 2H, NH_2_), 2.68 (s, 3H, CH_3_), 2.41 (s, 3H, CH_3_). ^13^C NMR (DMSO-*d*_6_) δ 167.9, 156.4, 155.9, 155.1, 142.8, 138.0, 137.9, 137.0, 136.6, 128.7 (2C), 127.3 (2C), 126.7 (2C), 123.9 (2C), 117.7, 117.0, 23.1, 21.1. IR 3338 (stretching -NH), 3261 (stretching -NH), 1651 (stretching -C=O) cm^−1^. Elemental analysis calculated for C_21_H_21_N_5_O_4_S (439.49): %C, 57.39; %H, 4.82; %N, 15.94. Found: %C, 57.41; %H, 4.81; %N, 15.96. *m*/*z* 440.

##### 2-(6-(3-Bromophenyl)-2-methylnicotinoyl)-N-(4-sulfamoylphenyl)hydrazinecarboxamide (**5d**)

Following the general procedure, the title compound was prepared starting from **4d**. Yield 39%; M.p. >250 °C. ^1^H NMR (DMSO-*d*_6_) δ 10.24 (s, 1H, NH), 9.30 (s, 1H, NH), 8.48 (s, 1H, NH), 8.33 (s, 1H, Ar), 8.15 (d, *J* = 8.1 Hz, 1H, Ar), 7.98 (d, *J* = 8.0 Hz, 1H, Ar), 7.73 (d, *J* = 8.8 Hz, 2H, Ar), 7.64–7.70 (m, 3H, Ar), 7.49 (t, *J* = 7.8 Hz, 1H, Ar), 7.20 (s, 2H, NH_2_), 6.75 (d, *J* = 8.8 Hz, 1H), 2.69 (s, 3H, CH_3_). ^13^C NMR (DMSO-*d*_6_) δ 167.7, 156.1, 154.5, 142.8, 140.2, 137.0, 136.9, 132.2, 131.0, 129.3, 129.2, 128.9, 126.7, 125.7, 122.4, 118.9, 117.7, 117.4, 115.2, 23.0. IR 315 (stretching -NH), 1644 (stretching -C=O) cm^−1^. Elemental analysis calculated for C_20_H_18_BrN_5_O_4_S (504.36): %C, 47.63; %H, 3.60; %N, 13.89. Found: %C, 47.63; %H, 3.59; %N, 13.91. *m*/*z* 505.

##### 2-(6-(4-Fluorophenyl)-2-methylnicotinoyl)-N-(4-sulfamoylphenyl)hydrazinecarboxamide (**5e**)

Following the general procedure, the title compound was prepared starting from **4e**. Yield 65%; M.p. >250 °C. ^1^H NMR (DMSO-*d*_6_) δ 10.21 (s, 1H, NH), 9.30 (s, 1H, NH), 8.47 (s, 1H, NH), 8.18–8.22 (m, 2H, Ar), 7.89–7.96 (m, 2H, Ar), 7.73 (d, *J* = 8.9 Hz, 2H, Ar), 7.66 (d, *J* = 8.8 Hz, 2H, Ar), 7.34 (t, *J* = 8.9 Hz, 2H, Ar), 7.20 (s, 2H, NH_2_), 2.68 (s, 3H, CH_3_). ^13^C NMR (DMSO-*d*_6_) δ 167.8, 163.9, 162.3, 156.0, 155.2, 155.1, 142.8, 137.0, 136.8, 134.4 (2C), 129.0, 128.9, 128.2, 126.7, 117.7, 116.8, 115.7, 115.6, 23.1. IR 3266 (stretching -NH), 1648 (stretching -C=O) cm^−1^. Elemental analysis calculated for C_20_H_18_FN_5_O_4_S (443.45): %C, 54.17; %H, 4.09; %N, 15.79. Found: %C, 54.16; %H, 4.09; %N, 15.77. *m*/*z* 444.

##### 2-(2-Methyl-6-(4-(trifluoromethyl)phenyl)nicotinoyl)-N-(4-sulfamoylphenyl)hydrazinecarboxamide (**5f**)

Following the general procedure, the title compound was prepared starting from **4f**. Yield 34%; M.p. >250 °C. ^1^H NMR (DMSO-*d*_6_) δ 10.26 (s, 1H, NH), 9.50 (s, 1H, NH), 8.58 (s, 1H, NH), 8.35 (d, *J* = 7.8 Hz, 2H, Ar), 8.02 (t, *J* = 10.7 Hz, 2H, Ar), 7.88 (d, *J* = 7.9 Hz, 2H, Ar), 7.74 (d, *J* = 8.5 Hz, 2H, Ar), 7.67 (d, *J* = 7.9 Hz, 2H, Ar), 7.20 (s, 2H, NH_2_), 2.71 (s, 3H, CH_3_). ^13^C NMR (DMSO-*d*_6_) δ 167.7, 156.3, 155.1, 154.6, 142.9, 141.7, 136.9, 129.6, 129.4, 129.3, 127.5 (2C), 125.8, 125.7 (2C), 125.7, 125.1, 123.3, 117.8, 117.6, 23.0. IR 3284 (stretching -NH), 1674 (stretching -C=O) cm^−1^. Elemental analysis calculated for C_21_H_18_F_3_N_5_O_4_S (493.46): %C, 51.11; %H, 3.68; %N, 14.19. Found: %C, 51.10; %H, 3.68; %N, 14.17. *m*/*z* 494.

##### 2-(2-Methyl-6-(3,4,5-trimethoxyphenyl)nicotinoyl)-N-(4-sulfamoylphenyl)hydrazinecarboxamide (**5g**)

Following the general procedure, the title compound was prepared starting from **4g**. Yield 66%; M.p. >250 °C. ^1^H NMR (DMSO-*d*_6_) δ 10.20 (s, 1H, NH), 9.31 (s, 1H, NH), 8.46 (s, 1H, NH), 7.95 (d, *J* = 8.2 Hz, 1H, Ar), 7.88 (d, *J* = 8.1 Hz, 1H, Ar), 7.73 (d, *J* = 8.9 Hz, 2H, Ar), 7.66 (d, *J* = 8.9 Hz, 2H, Ar), 7.44 (s, 2H, Ar), 7.20 (s, 2H, NH_2_), 3.89 (s, 6H, CH_3_), 3.73 (s, 3H, CH_3_), 2.68 (s, 3H, CH_3_). ^13^C NMR (DMSO-*d*_6_) δ 168.4, 156.4, 156.3, 155.5, 153.7, 143.3, 139.4, 137.5, 137.0, 136.7, 133.9, 129.8, 128.4 (2C), 127.2, 118.2, 117.5, 115.7, 104.7, 60.6, 56.5 (2C), 23.7. IR 3312 (stretching -NH), 1659 (stretching -C=O) cm^−1^. Elemental analysis calculated for C_23_H_25_N_5_O_7_S (515.54): %C, 53.58; %H, 4.89; %N, 13.58. Found: %C, 53.60; %H, 4.89; %N, 13.59. *m*/*z* 516.

##### 2-(6-(3,4-Dimethoxyphenyl)-2-methylnicotinoyl)-N-(4-sulfamoylphenyl)hydrazinecarboxamide (**5h**)

Following the general procedure, the title compound was prepared starting from **4h**. Yield 74%; M.p. >250 °C. ^1^H NMR (DMSO-*d*_6_) δ 10.17 (s, 1H, NH), 9.32 (s, 1H, NH), 8.47 (s, 1H, NH), 7.88 (s, 2H, Ar), 7.71–7.75 (m, 4H, Ar), 7.66 (d, *J* = 8.8 Hz, 2H, Ar), 7.19 (s, 2H, NH_2_), 7.08 (d, *J* = 8.6 Hz, 1H, Ar), 3.87 (s, 3H, CH_3_), 3.83 (s, 3H, CH_3_), 2.67 (s, 3H, CH_3_). ^13^C NMR (DMSO-*d*_6_) δ 167.9, 156.1, 155.8, 155.0, 150.2, 148.9, 142.8, 137.0, 136.5, 130.6, 127.4 (2C), 126.8, 126.7, 119.6, 117.7, 116.3, 111.7, 110.0, 55.6 (2C), 23.2. IR 3303 (stretching -NH), 1643 (stretching -C=O) cm^−1^. Elemental analysis calculated for C_22_H_23_N_5_O_6_S (485.51): %C, 54.76; %H, 4.60; %N, 17.42. Found: %C, 54.77; %H, 4.59; %N, 17.44. *m*/*z* 486.

##### 2-(2-Methyl-6-(4-(methylsulfonamido)phenyl)nicotinoyl)-N-(4-sulfamoylphenyl)hydrazinecarboxamide (**5i**)

Following the general procedure, the title compound was prepared starting from **4i**. Yield 60%; M.p. >250 °C. ^1^H NMR (DMSO-*d*_6_) δ 10.21 (s, 1H, NH), 10.11 (s, 1H, NH), 9.30 (s, 1H, NH), 8.47 (s, 1H, NH), 8.29 (t, *J* = 2.0 Hz, 1H, Ar), 7.94 (d, *J* = 9.7 Hz, 1H, Ar), 7.82 (d, *J* = 8.0 Hz, 1H, Ar), 7.72–7.78 (m, 4H, Ar), 7.66 (d, *J* = 8.5 Hz, 2H, Ar), 7.43 (t, *J* = 7.9 Hz, 1H, Ar), 7.20 (s, 2H, NH_2_), 2.68 (s, 3H, CH_3_), 2.08 (s, 3H, CH_3_). ^13^C NMR (DMSO-*d*_6_) δ 167.8, 156.1, 155.5, 155.1, 142.8, 142.7, 137.0, 136.8 (2C), 136.2 (2C), 127.4, 126.7 (2C), 126.1 (2C), 117.7, 117.0 (2C), 35.3, 23.1. IR 3235 (stretching -NH), 1652 (stretching -C=O) cm^−1^. Elemental analysis calculated for C_21_H_22_N_6_O_6_S_2_ (518.47): %C, 48.64; %H, 4.28; %N, 16.21. Found: %C, 48.65; %H, 4.29; %N, 16.21. *m*/*z* 519.

##### 2-(6-(4-Acetamidophenyl)-2-methylnicotinoyl)-N-(4-sulfamoylphenyl)hydrazinecarboxamide (**5j**)

Following the general procedure, the title compound was prepared starting from **45**. Yield 78%; M.p. >250 °C. ^1^H NMR (DMSO-*d*_6_) δ 10.22 (s, 1H, NH), 9.30 (s, 1H, NH), 8.47 (s, 1H, NH), 8.14–8.18 (m, 2H, Ar), 7.92 (d, *J* = 8.0 Hz, 2H, Ar), 7.73 (d, *J* = 9.0 Hz, 2H, Ar), 7.66 (d, *J* = 8.8 Hz, 2H, Ar), 7.55 (d, *J* = 8.8 Hz, 2H, Ar), 7.20 (s, 2H, NH_2_), 3.00 (s, 3H, CH_3_), 2.68 (s, 3H, CH_3_). ^13^C NMR (DMSO-*d*_6_) δ 168.5, 167.9, 156.2, 156.0, 155.1, 142.8, 139.8, 138.4, 137.0, 136.8, 129.2 (2C), 128.4, 126.7 (2C), 121.4, 120.1, 117.7, 117.4, 117.0, 24.0, 23.0. IR 3278 (stretching -NH), 1647 (stretching -C=O) cm^−1^. Elemental analysis calculated for C_22_H_22_N_6_O_5_S (482.51): %C, 54.76; %H, 4.60; %N, 17.42. Found: %C, 54.76; %H, 4.59; %N, 17.41. *m*/*z* 483.

### 3.2. Carbonic Anhydrase Inhibition

An Applied Photophysics stopped-flow instrument was used for assaying the CA-catalyzed CO_2_ hydration activity using the Khalifah procedure [44]. The indicator used was phenol red (0.2 mM), the absorbance maximum was 557 nm, and the buffer was 20 mM Hepes (pH 7.5), whereas 20 mM sodium sulfate was employed to maintain a constant ionic strength. The initial rates of the CA-catalyzed CO_2_ hydration reaction were monitored over 10–100 s, working at CO_2_ concentrations of 1.7 to 17 mM. Six traces of the initial 5–10% of the reaction were used for each inhibitor for the assessment of the initial velocity. The uncatalyzed rates were subtracted from the observed total rates. Standard acetazolamide and test compound stock solutions (0.1 mM) were prepared in a 10% DMSO aqueous solution and diluted to 0.01 nM with the assay buffer. Inhibitor and enzyme solutions were preincubated together for 15 min, to ensure the formation of the E–I complex. The inhibition constants were obtained by non-linear least squares using the Cheng–Prusoff equation, as reported earlier [45,46,47], and represent the mean of at least three different determinations. All CA isoforms were recombinant ones obtained in-house, as reported earlier [48,49]. Their concentrations in the assay system were 5.7–11.9 nM.

### 3.3. Molecular Docking

Molecular docking simulations were carried out by means of the GOLD software (2024.1 CSD Release) [50]. The crystal structures of hCA I (PDB ID 3W6H), hCA II (PDB ID 3HS4), hCA IX (PDB ID 3IAI) and hCA XII (PDB ID 1JD0), all in complex with AAZ, were used as 3D coordinates. Protein and ligand preparation protocols, as well as the docking simulation procedures, were performed as previously described in our earlier work [51]. The top-ranked docking pose was selected for analysis and graphical representation.

## 4. Conclusions

CAs are well known to be involved in several physiological and pathological processes including glaucoma, obesity, osteoporosis, cancer, high-altitude sickness, epilepsy, neuropathic pain, and sleep apnea, making them highly studied targets in medicinal chemistry. The potential of hydrazidoureido or hydrazidothioureido benzene sulfonamide derivatives as isoform-selective CAIs led us to synthesize a novel series of 2-(aryl)-2-methylnicotinoyl)-N-(4-sulfamoylphenyl)hydrazinecarboxamide **5** bearing the 6-arylpyridine tail to evaluate their effect on four hCA isozymes (hCAs I, II, IX and XII). Kinetic studies revealed that all compounds exhibited strong inhibition against the tested isozymes in the nanomolar range. All compounds showed a good inhibition profile toward the tumor-associated isoform hCA XII, showing inhibition values ranging from 5.8 nM to 56.2 nM; compound **5f**, compound **5i** and compound **5j** also demonstrated good selectivity toward this isoform. Docking studies revealed that the high hCA XII selectivity of **5f**, **5i**, and **5j** arises from the different orientation of their tails with respect to the other investigated hCA isoforms. Furthermore, compounds **5f**, **5i** and **5j** showed favorable pharmacokinetic properties in SwissADME predictions. Based on these findings, these compounds could constitute a starting point for the development of isoform-selective hCAIs, particularly for highly effective and selective molecules targeting cytosolic hCA II and tumor-associated hCA XII.

## Figures and Tables

**Table 1:** Inhibition activity of compounds **5a-j** on hCA I, hCA II, hCA IX and hCA XII, using Acetozolamide (AAZ) as a reference compound.

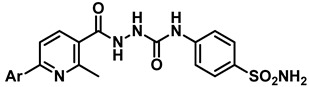					
		K_i_ (nM) *			
Compound	Ar	CA I	CA II	CA IX	CA XII
**5a**	3,5-diCF_3_ phenyl	298.2	9.3	195.7	8.9
**5b**	2-Benzofuryl	77.5	9.7	87.4	47.7
**5c**	3-CH_3_ phenyl	41.4	7.1	145.0	6.9
**5d**	3-Br phenyl	21.4	5.5	22.7	56.2
**5e**	4-F phenyl	32.5	6.4	92.7	9.4
**5f**	4-CF_3_ phenyl	73.8	223.0	94.1	5.8
**5g**	3,4,5-triOCH_3_ phenyl	34.2	34.6	90.3	8.5
**5h**	3,4-diOCH_3_ phenyl	74.4	3.8	317.1	8.8
**5i**	*N*-4-methanesulfonamide	750.4	44.2	474.4	7.8
**5j**	*N*-4-acetamide	90.0	37.2	90.4	8.7
**AAZ**		250.0	12.0	25.0	20.8

* K_i_ values are reported as the mean of three independent experiments. Errors are in the range of ±5–10% of the reported values.

**Table 2:** Predicted drug-like properties of selected compounds **5f**, **5i** and **5j**.

Compound	Solubility (ESOL)	Consensus LogP	H-Bond Acceptors	H-Bond Donors	P-gp Substrate	BB Permeation	Bioavailability Score
**5f**	Moderately soluble	2.66	9	4	No	No	0.55
**5i**	Soluble	0.86	8	5	No	No	0.17
**5j**	Soluble	1.25	7	5	No	No	0.55

## Data Availability

The original contributions presented in this study are included in the article/Supplementary Material. Further inquiries can be directed to the corresponding author.

## Supplementary Materials

The following supporting information can be downloaded at https://www.mdpi.com/article/10.3390/ph19020290/s1, S1: ^1^H and ^13^C NMR spectra of the final compounds **5a-j**; S2: IR spectra of the final compounds **5a-j**.

## Abbreviations

The following abbreviations are used in this manuscript:

CA	Carbonic anhydrase
HIF-1α	Hypoxia-inducible factor alpha
VHL	von Hippel–Lindau
DMF-DMA	Dimethylformamidedimethyl acetal
DMF	Dimethylformamide
