# Supplementary material for: Synthesis of Hydrazidoureidobenzensulfonamides Incorporating a Nicotinoyl Tail and Their Carbonic Anhydrase I, II, IX and XII Inhibitory Activity
Source: Pharmaceuticals (Basel). 2026 Feb 9;19(2):290. doi: 10.3390/ph19020290 (PMC12943707; doi:10.3390/ph19020290)

Synthesis of Hydrazidoureidobenzensulfonamides Incorporating a Nicotinoyl Tail and Their Carbonic Anhydrase I, II, IX and XII Inhibitory Activity

Alberto Deplano[1§], Davide Moi[1§], Serena Vittorio[2], Andrea Angeli[3], Claudiu T. Supuran[3], Valentina Onnis[1*]

[1]Dipartimento di Scienze della Vita e dell'Ambiente Università degli Studi di Cagliari, Cittadella universitaria di Monserrato, S.P. 8 CA, 09042 Monserrato, Italy; davide.moi@unica.it (D.M.); alberto.deplano@unica.it (A.D.)
[2]Dipartimento di Scienze Farmaceutiche, Università degli Studi di Milano, Via Mangiagalli 25, 20133 Milano, Italy; serena.vittorio@unimi.it
[3]Laboratorio di Chimica Bioinorganica, Polo Scientifico Neurofarba Department, Università degli Studi di Firenze, Room 188, Via della Lastruccia 3, Sesto Fiorentino, 50019 Florence, Italy; andrea.angeli@unifi.it (A.A.); claudiu.supuran@unifi.it (C.T.S.)

*Correspondence: valentina.onnis@unica.it; §These authors equally contribute.

Supporting informations content;
S1: ^{1}H and ^{13}C NMR spectra of the final compounds **5a-j**
S2: IR spectra of the final compounds **5a-j**

.

2-(6-(3,5-bis(trifluoromethyl)phenyl)-2-methylnicotinoyl)-N-(4-sulfamoylphenyl)hydrazinecarboxamide ***5a***

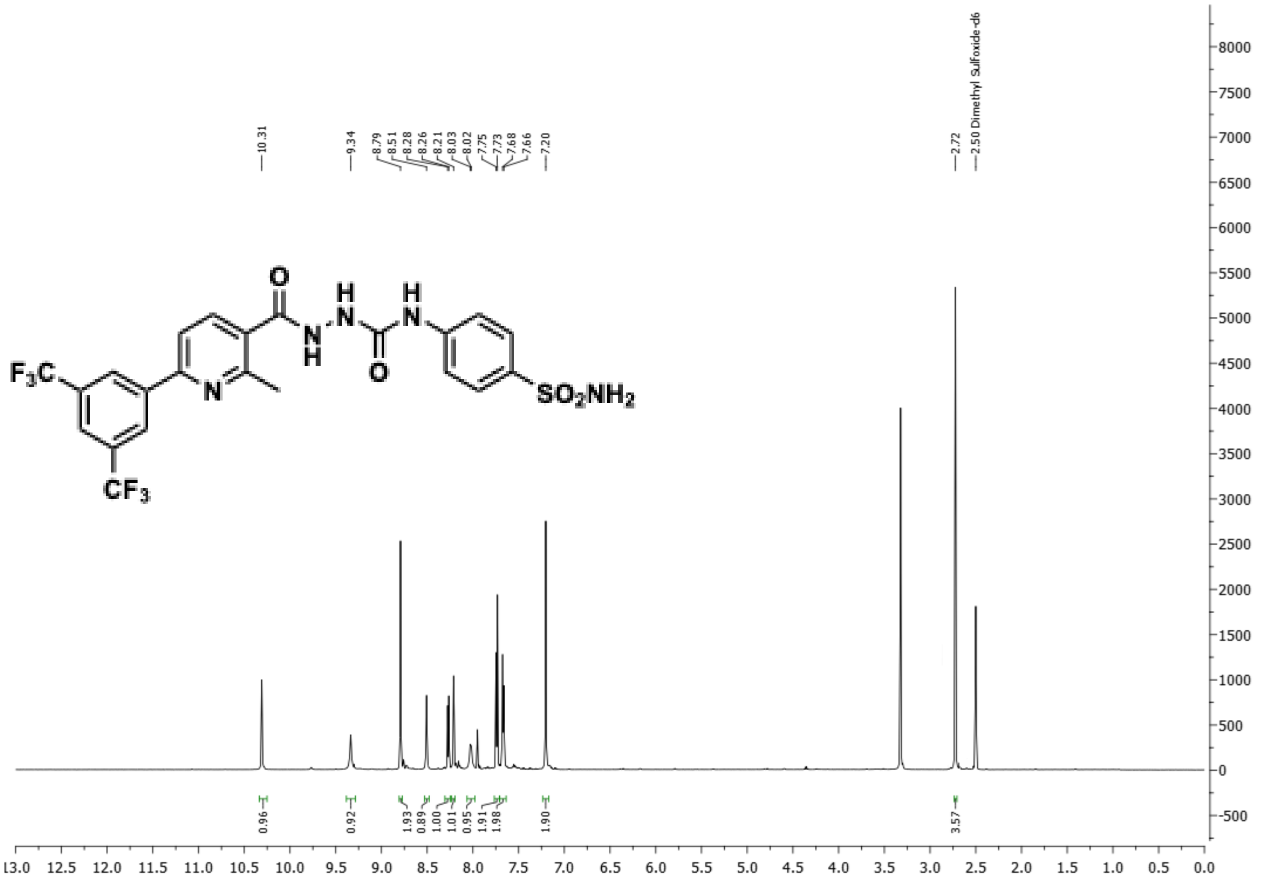

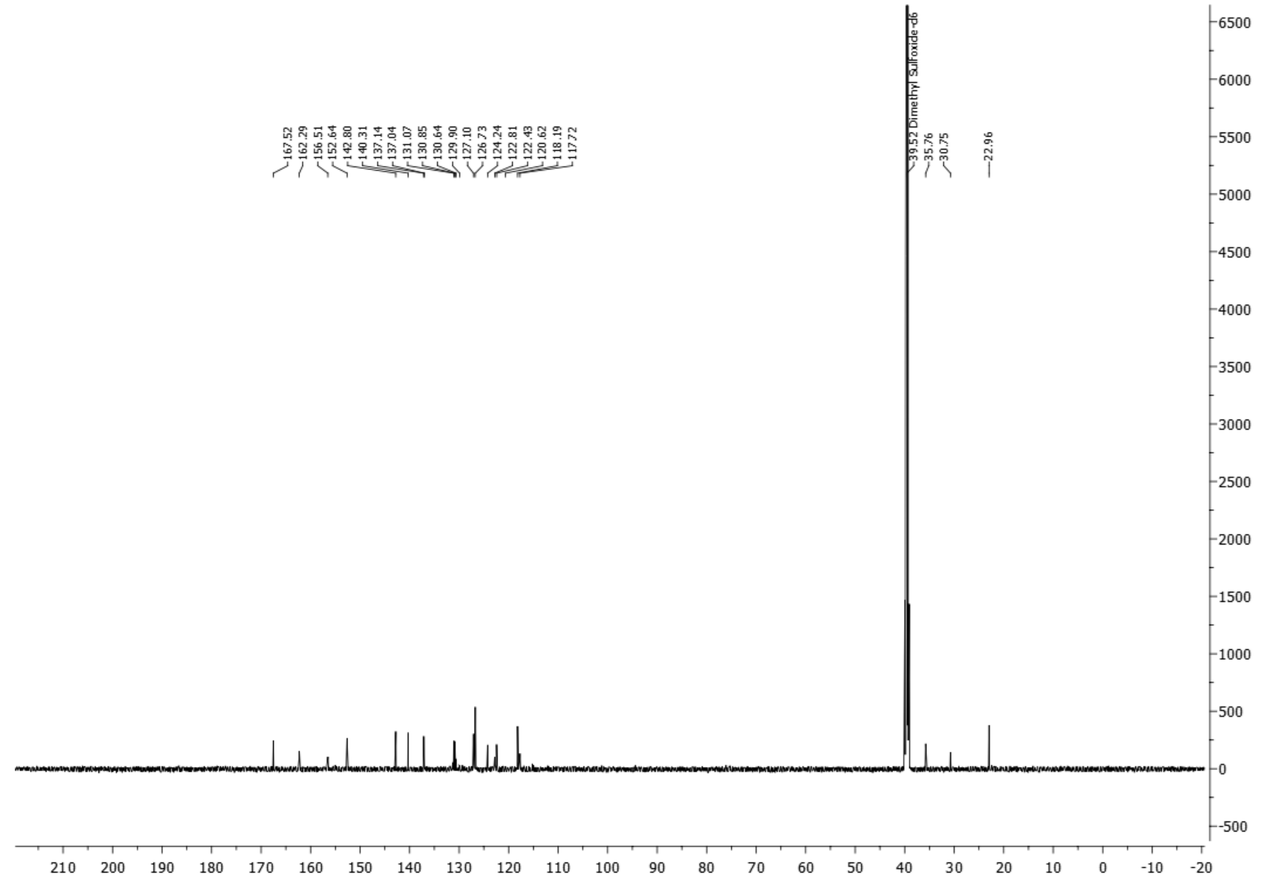

*2-(6-(benzofuran-2-yl)-2-methylnicotinoyl)-N-(4-sulfamoylphenyl)hydrazinecarboxamide **5b***

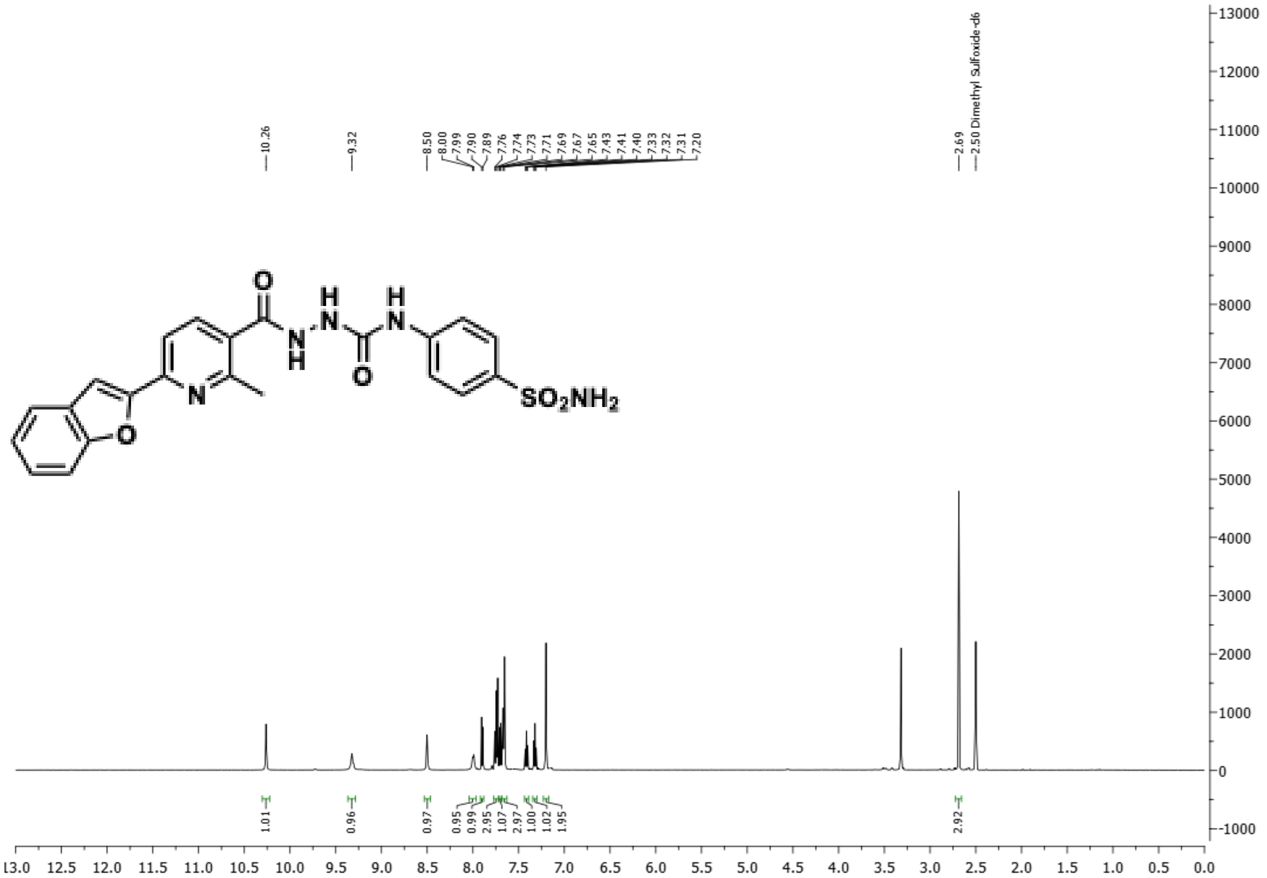

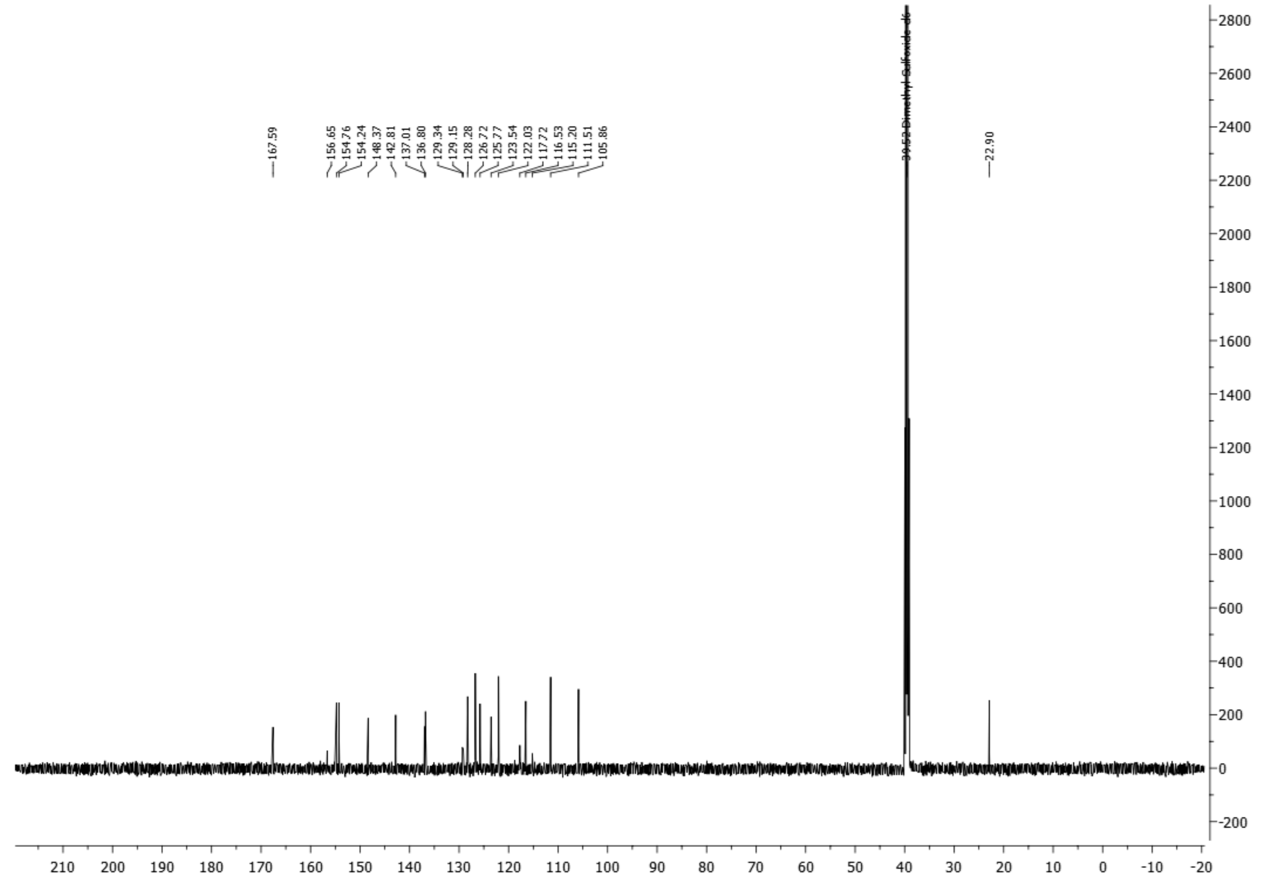

2-(2-methyl-6-(m-tolyl)nicotinoyl)-N-(4-sulfamoylphenyl)hydrazinecarboxamide ***5c***

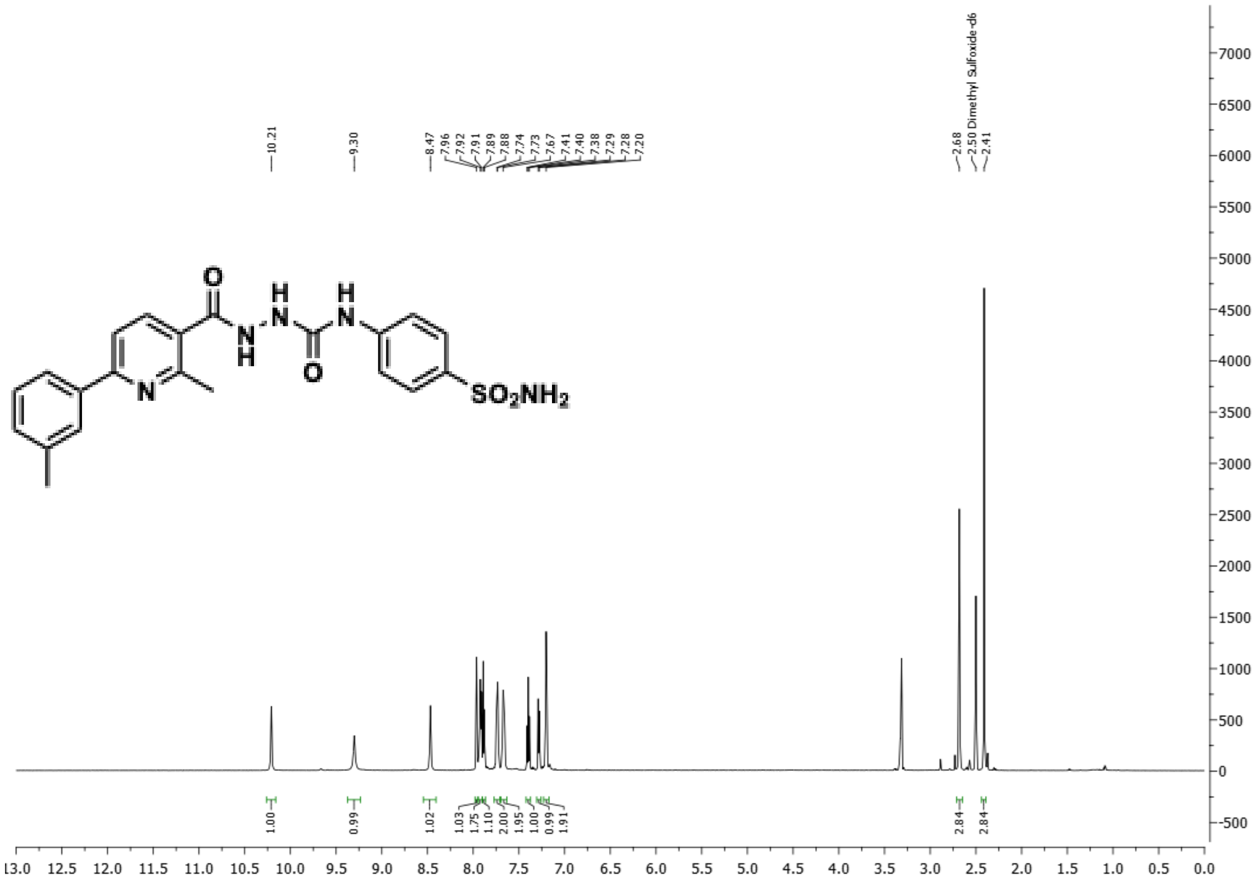

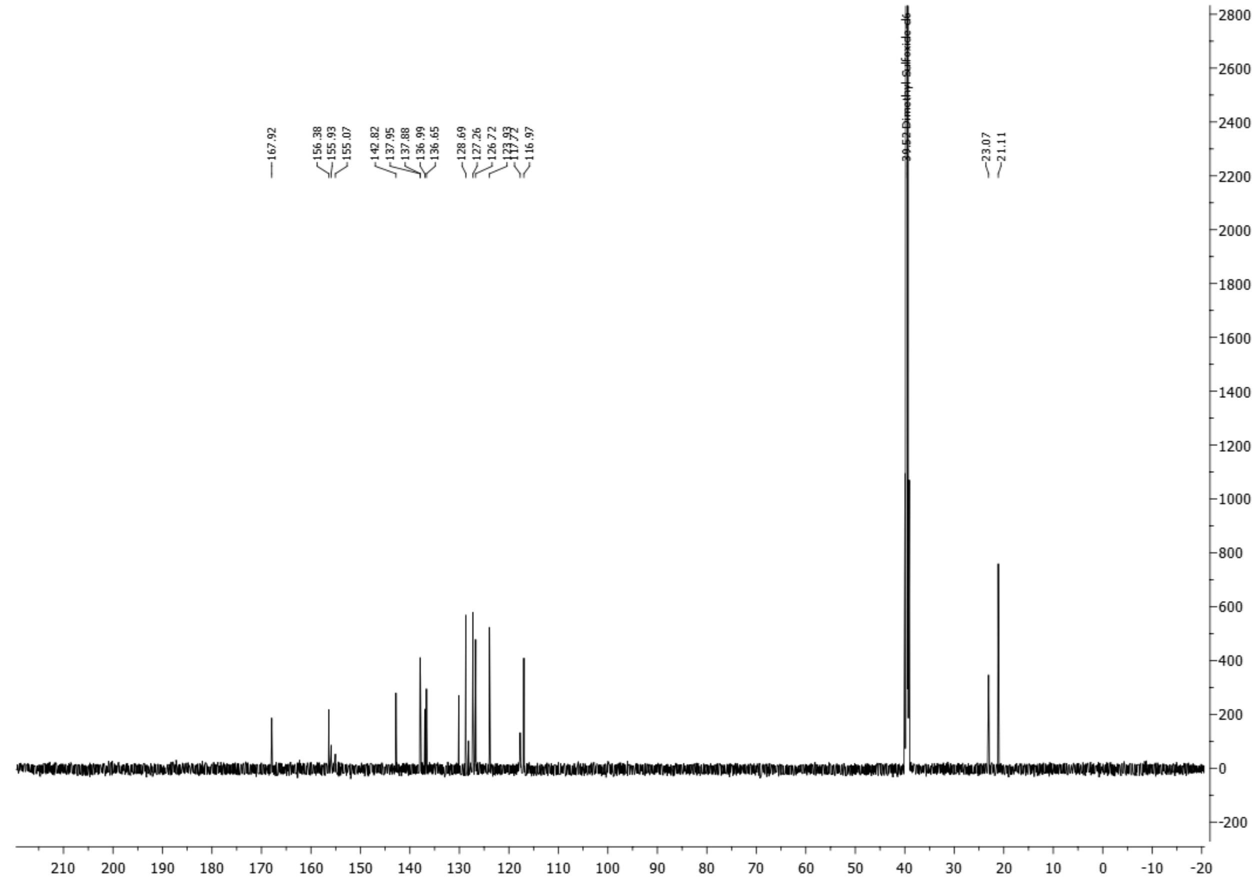

2-(6-(3-bromophenyl)-2-methylnicotinoyl)-N-(4-sulfamoylphenyl)hydrazinecarboxamide ***5d***

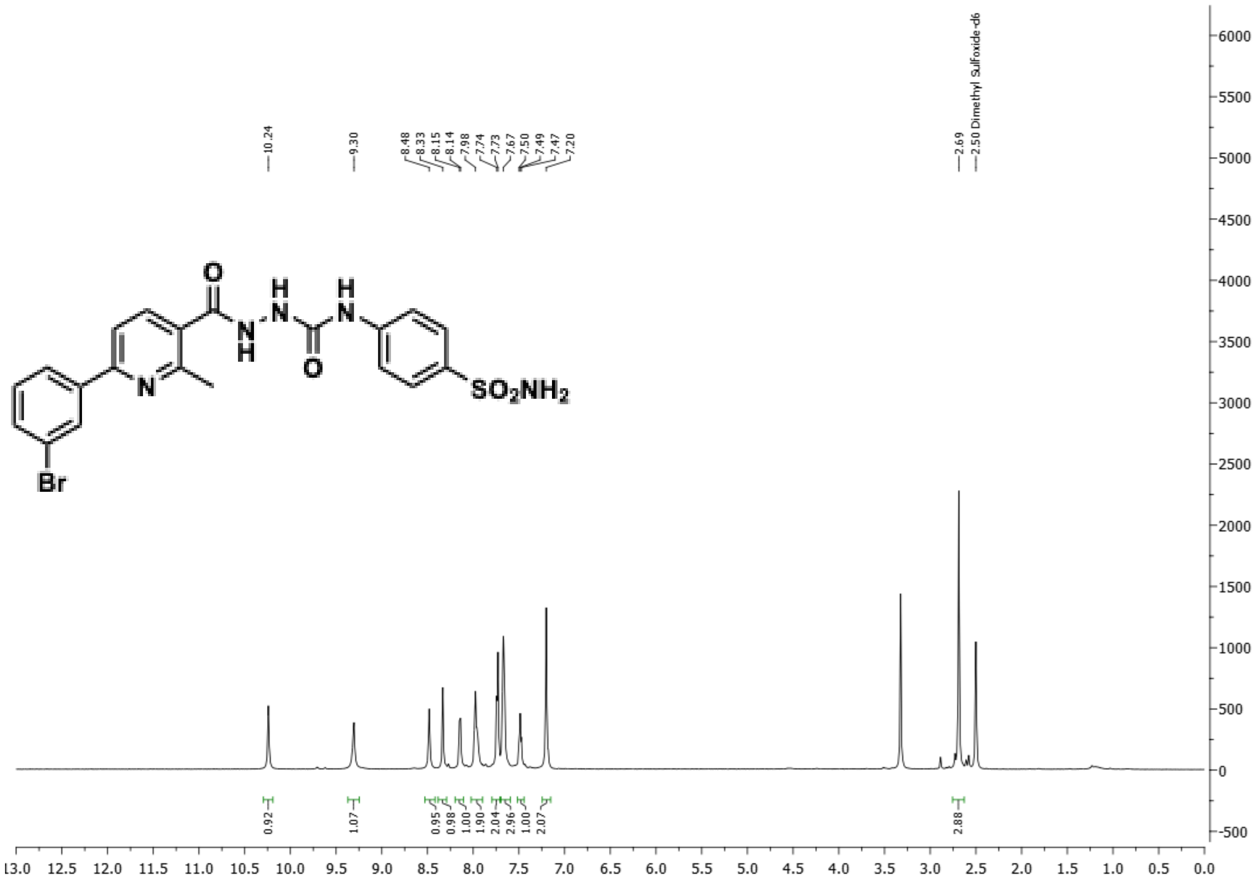

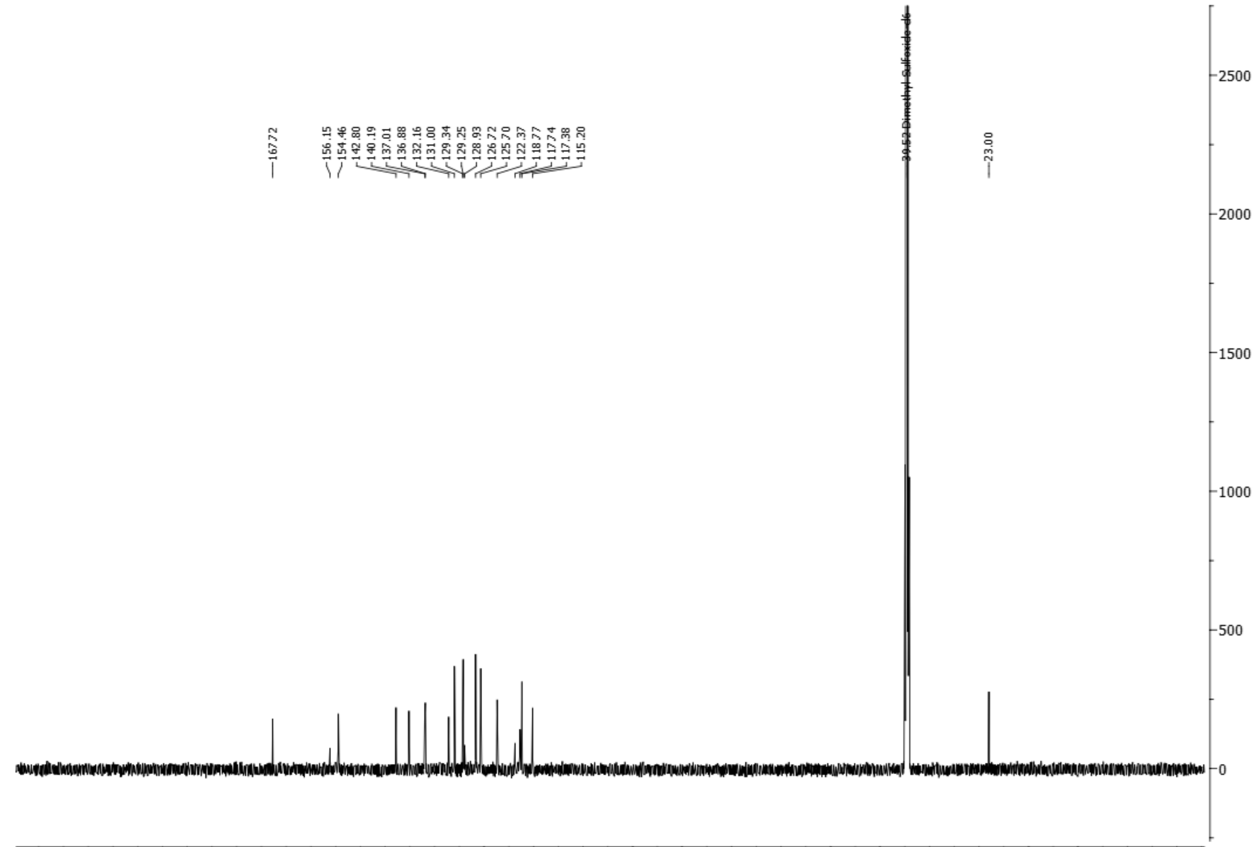

2-(6-(4-fluorophenyl)-2-methylnicotinoyl)-N-(4-sulfamoylphenyl)hydrazinecarboxamide ***5e***

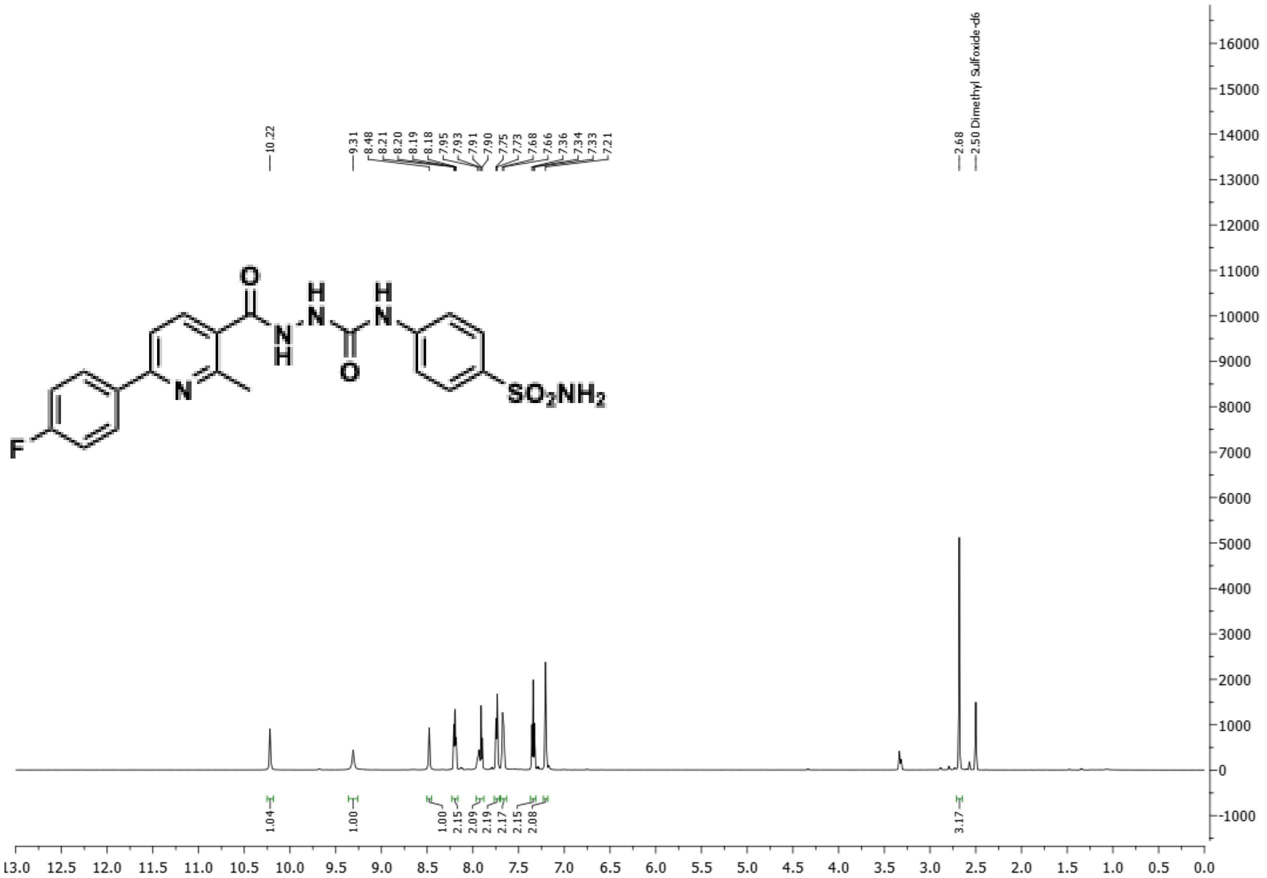

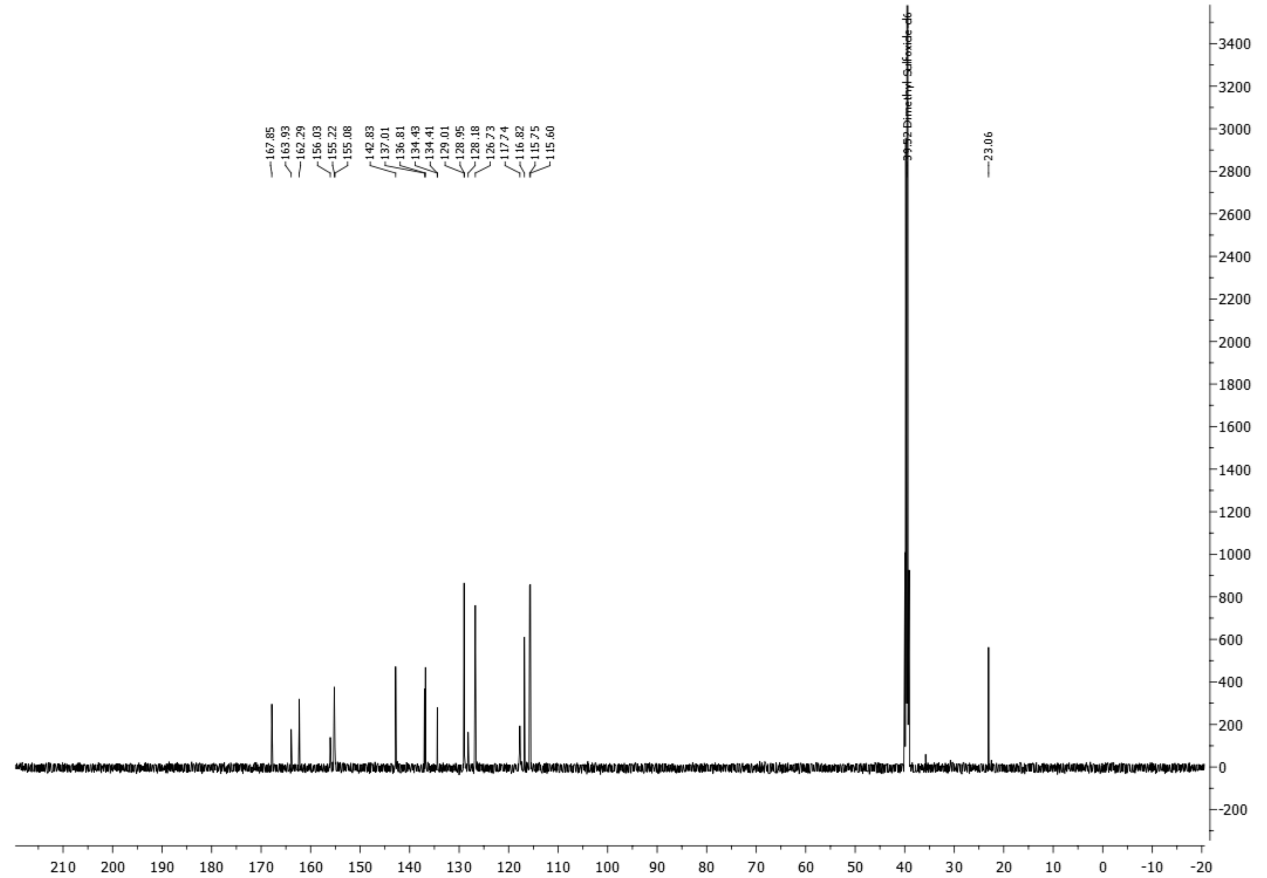

2-(2-methyl-6-(4-(trifluoromethyl)phenyl)nicotinoyl)-N-(4-sulfamoylphenyl)hydrazinecarboxamide ***5f***

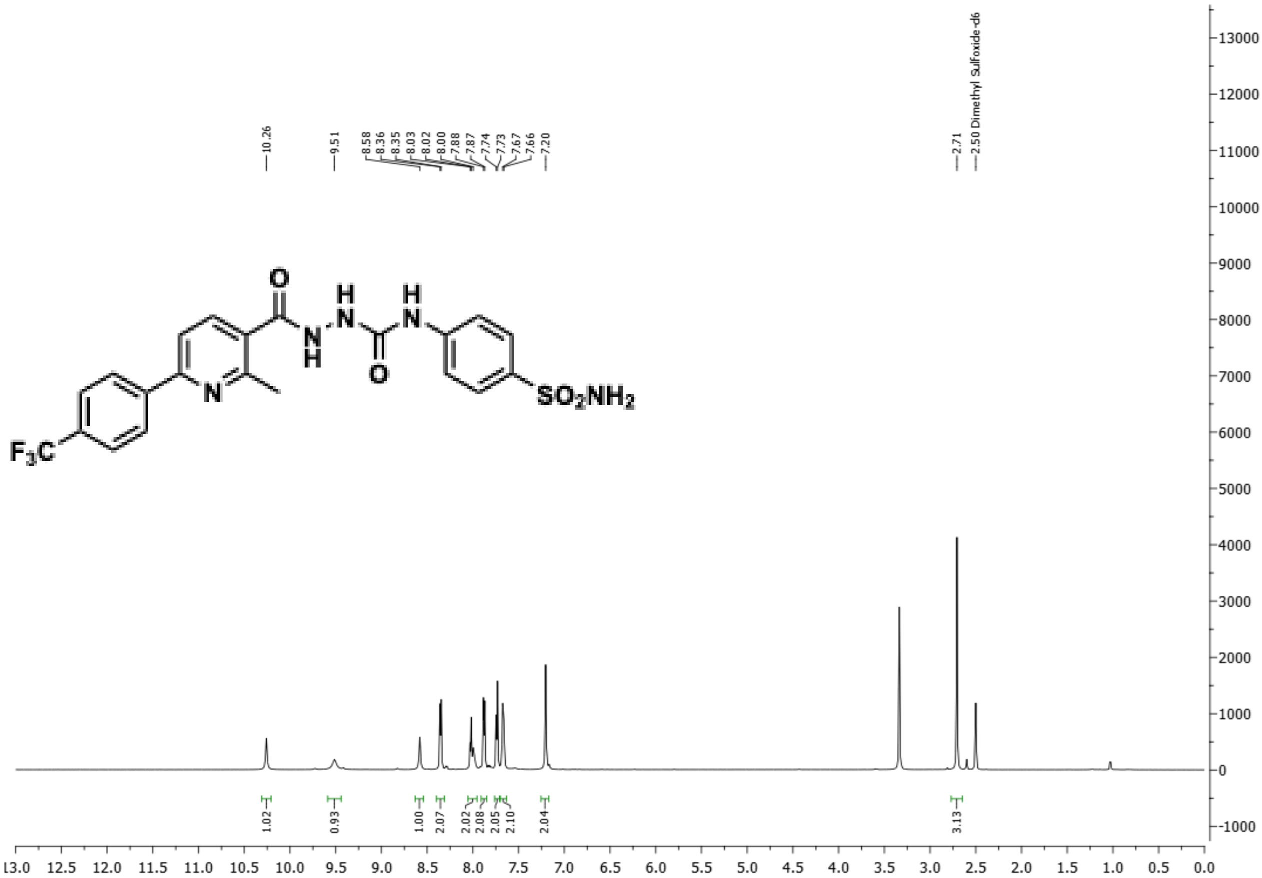

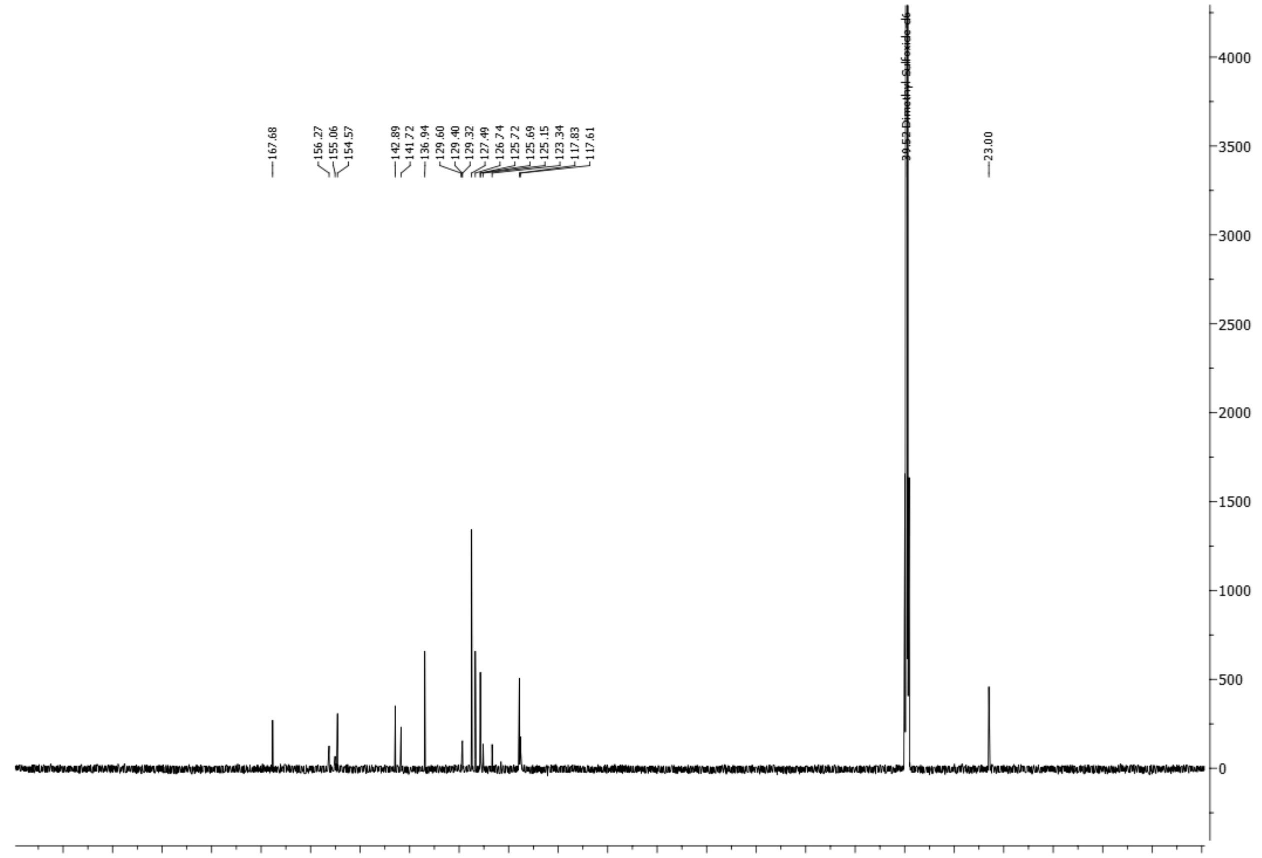

2-(2-methyl-6-(3,4,5-trimethoxyphenyl)nicotinoyl)-N-(4-sulfamoylphenyl)hydrazinecarboxamide ***5g***

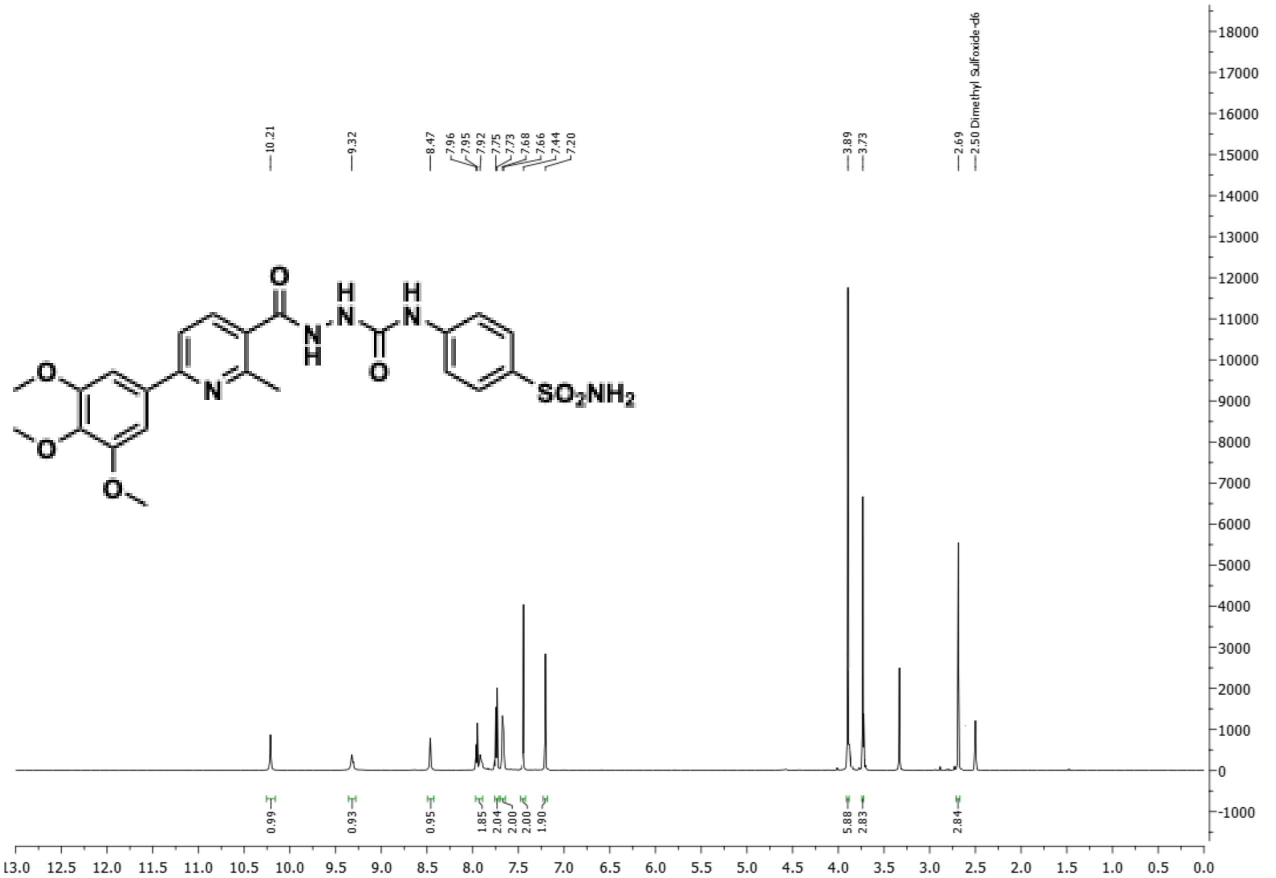

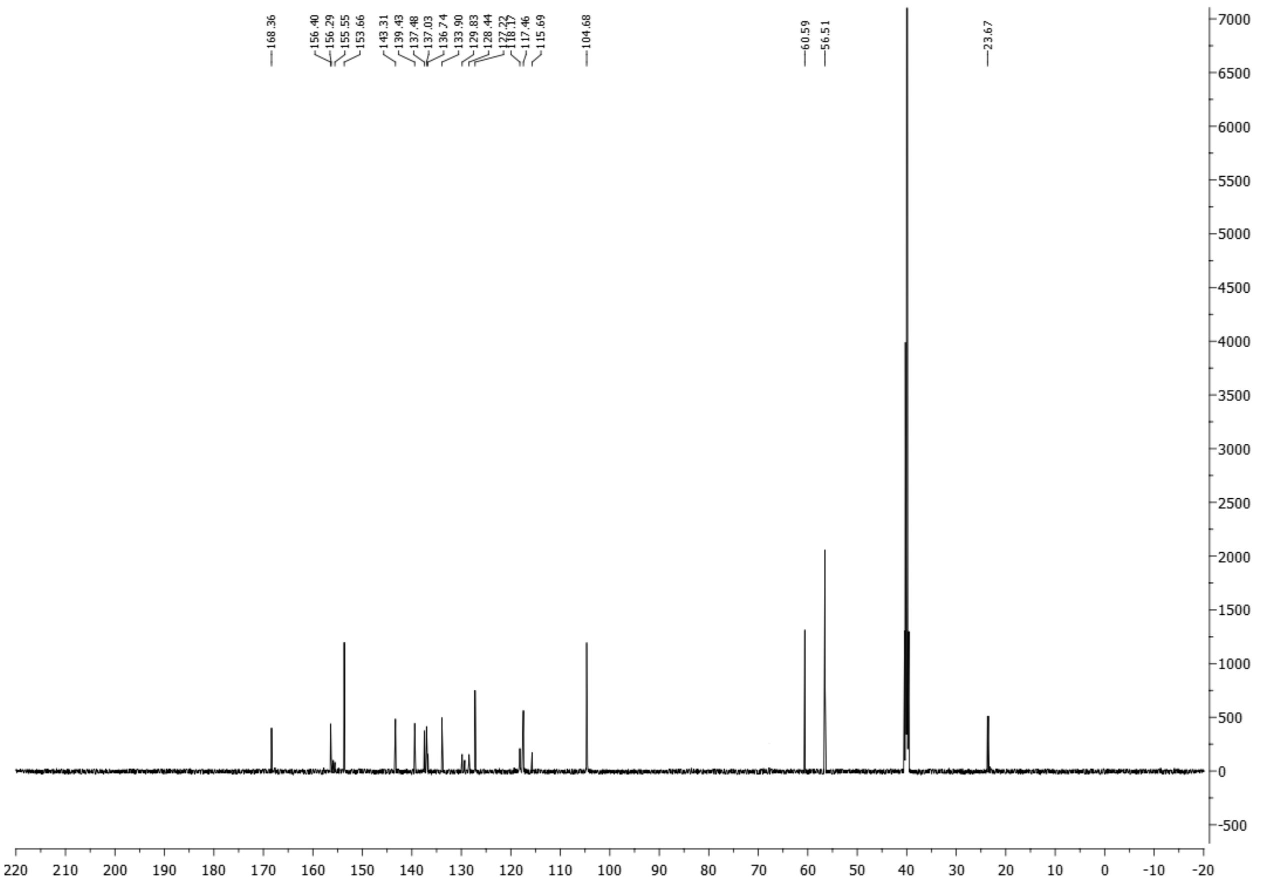

2-(6-(3,4-dimethoxyphenyl)-2-methylnicotinoyl)-N-(4-sulfamoylphenyl)hydrazinecarboxamide ***5h***

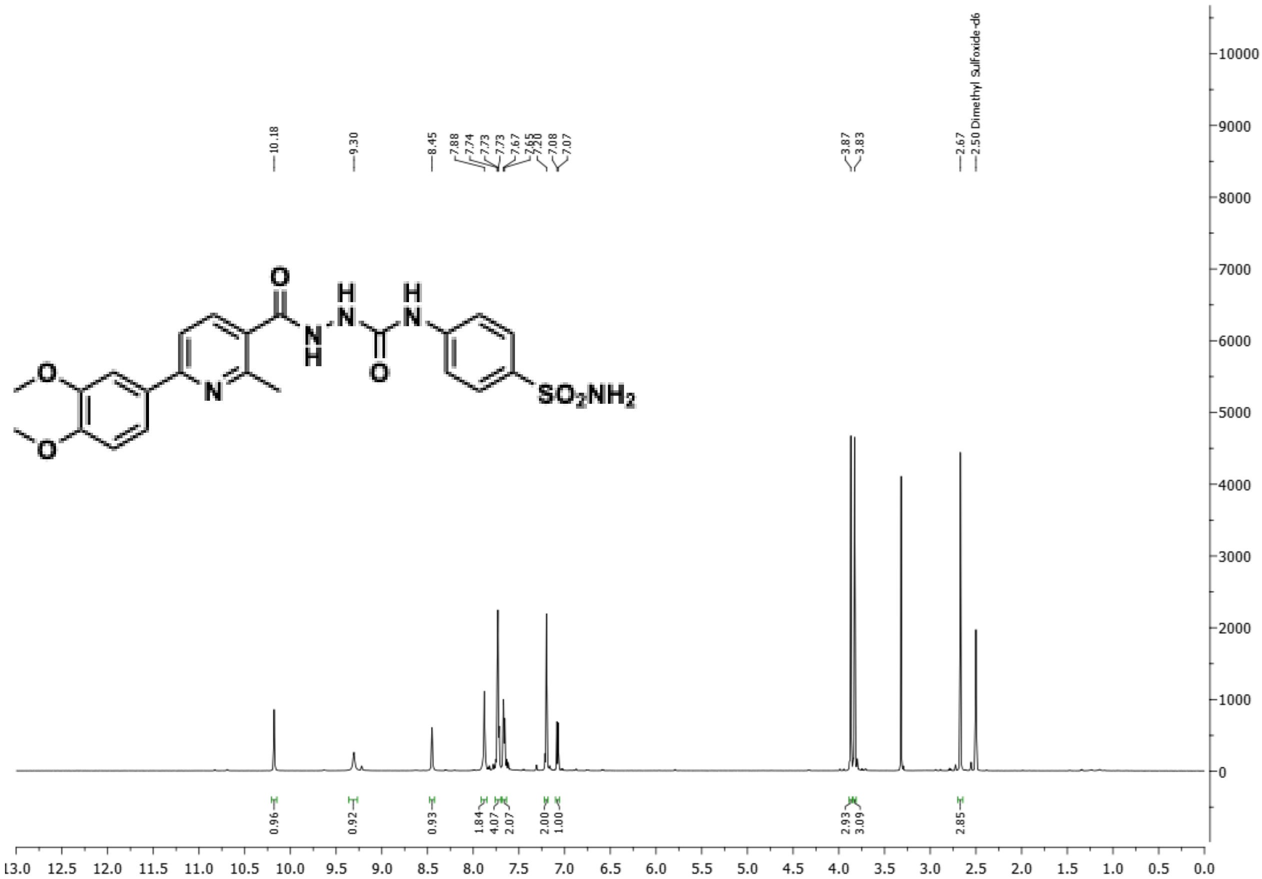

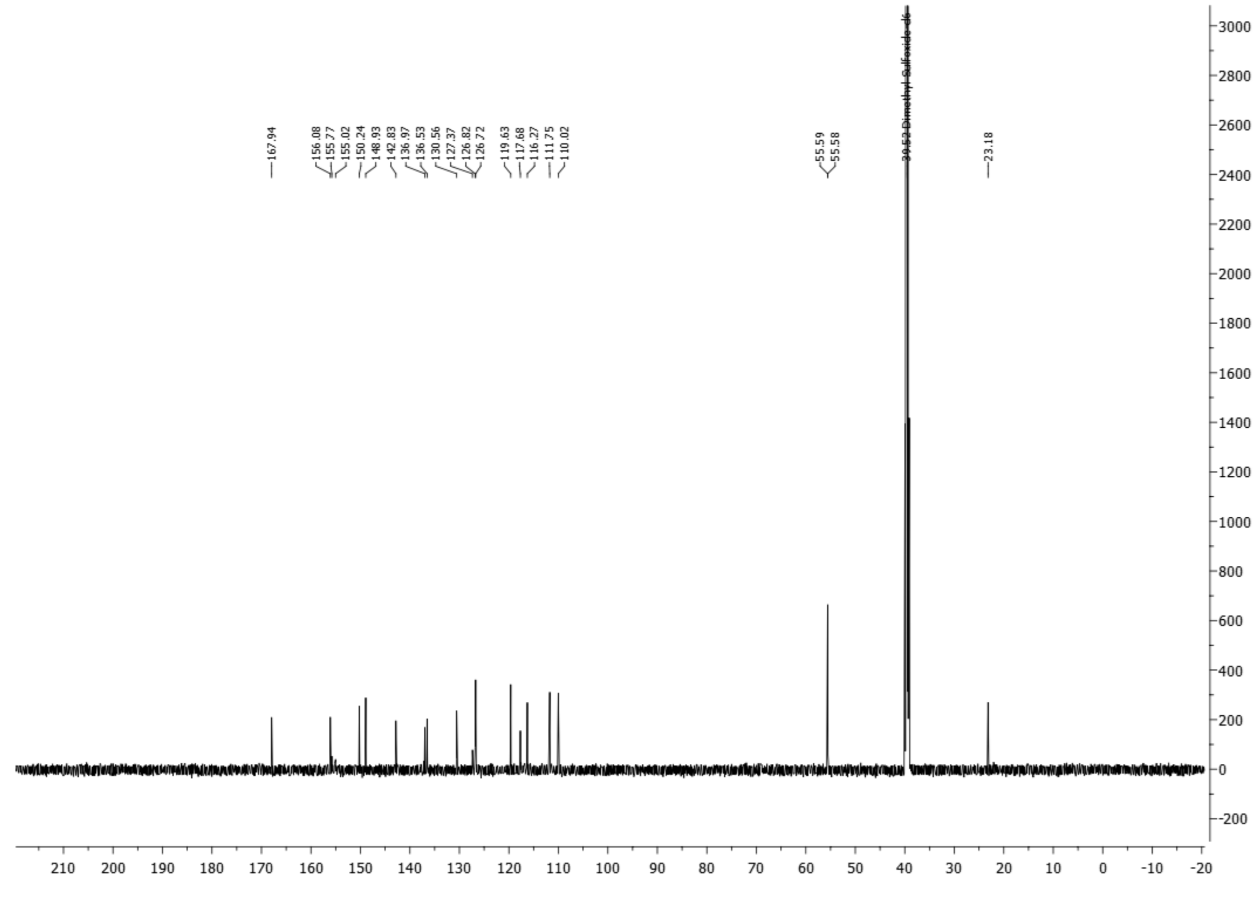

2-(2-methyl-6-(4-(methylsulfonamido)phenyl)nicotinoyl)-N-(4-sulfamoylphenyl)hydrazinecarboxamide ***5i***

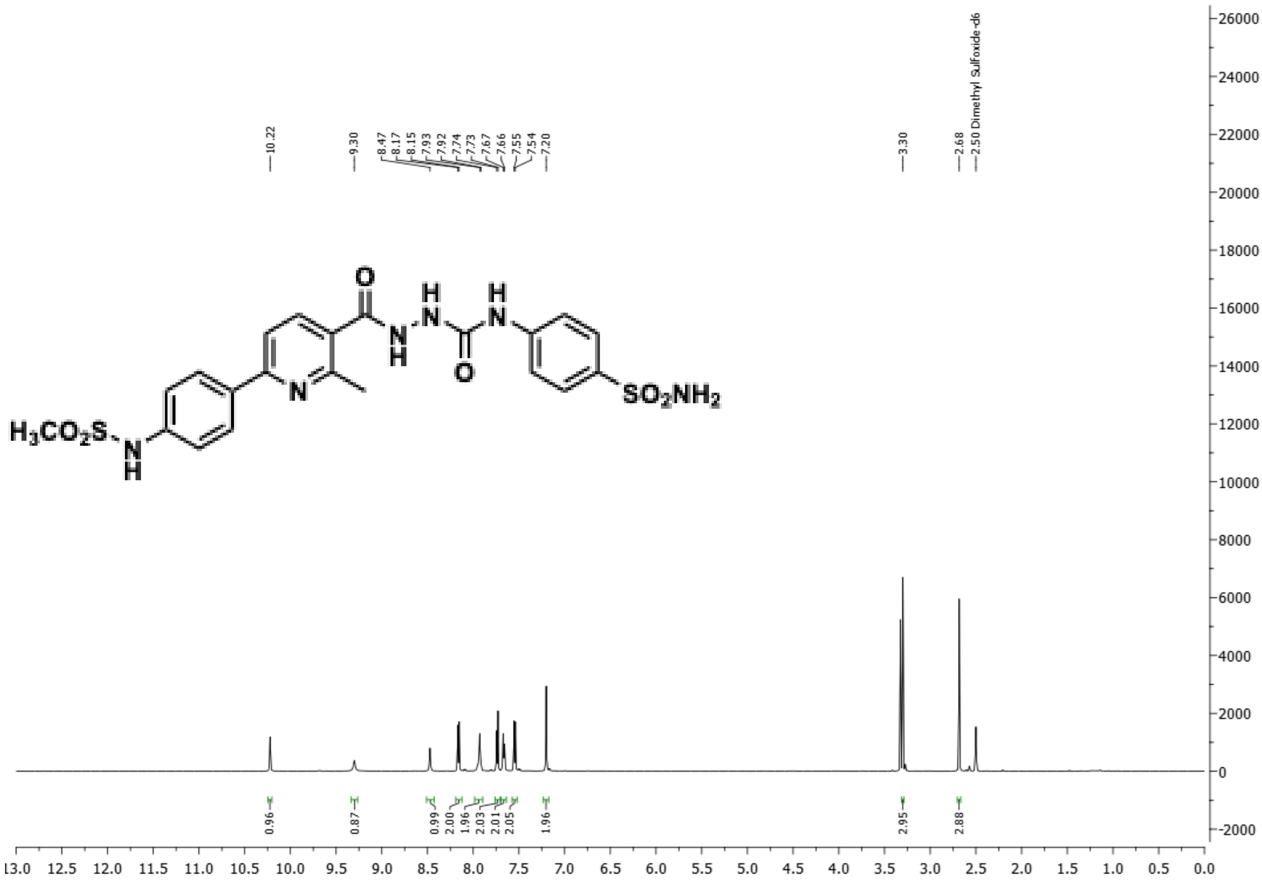

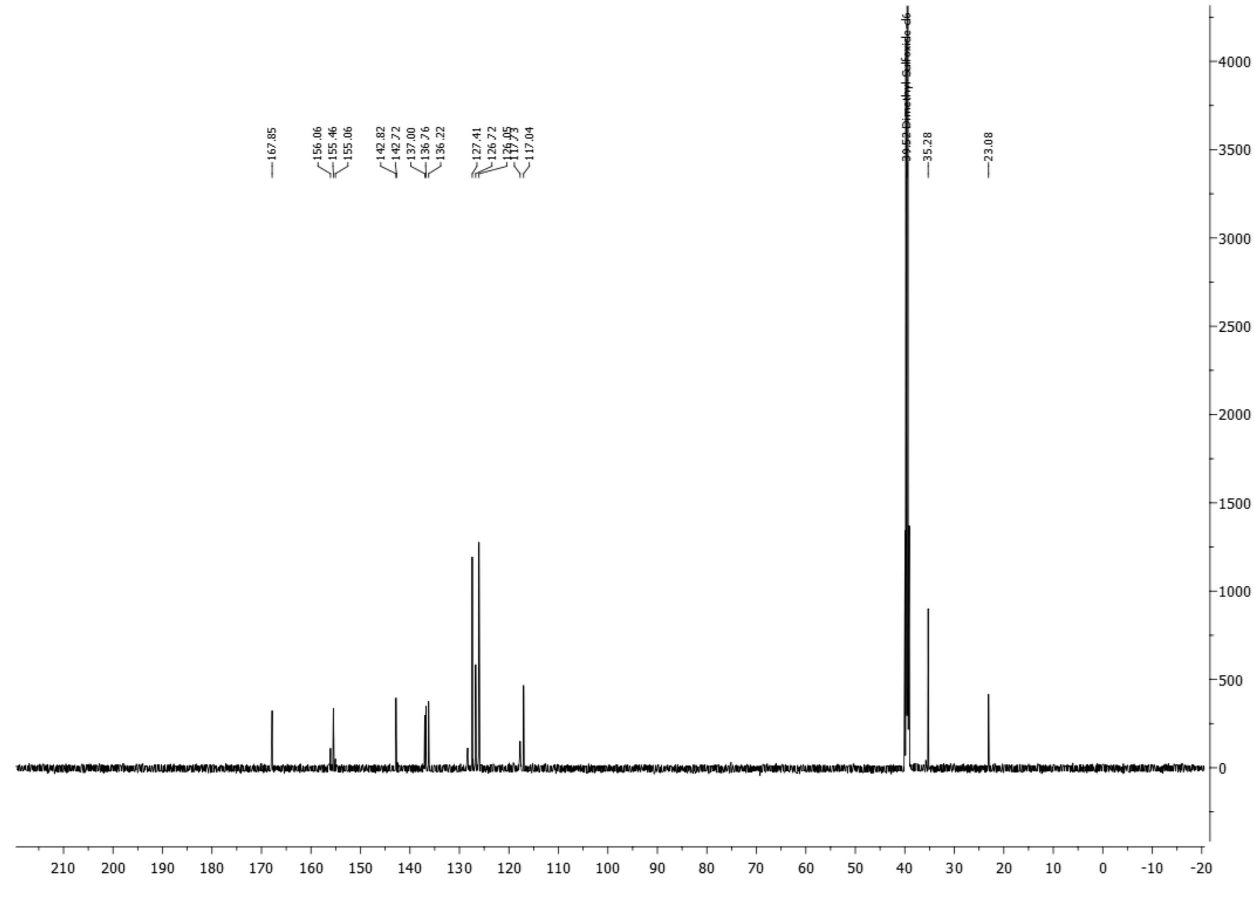

2-(6-(4-acetamidophenyl)-2-methylnicotinoyl)-N-(4-sulfamoylphenyl)hydrazinecarboxamide ***5j***

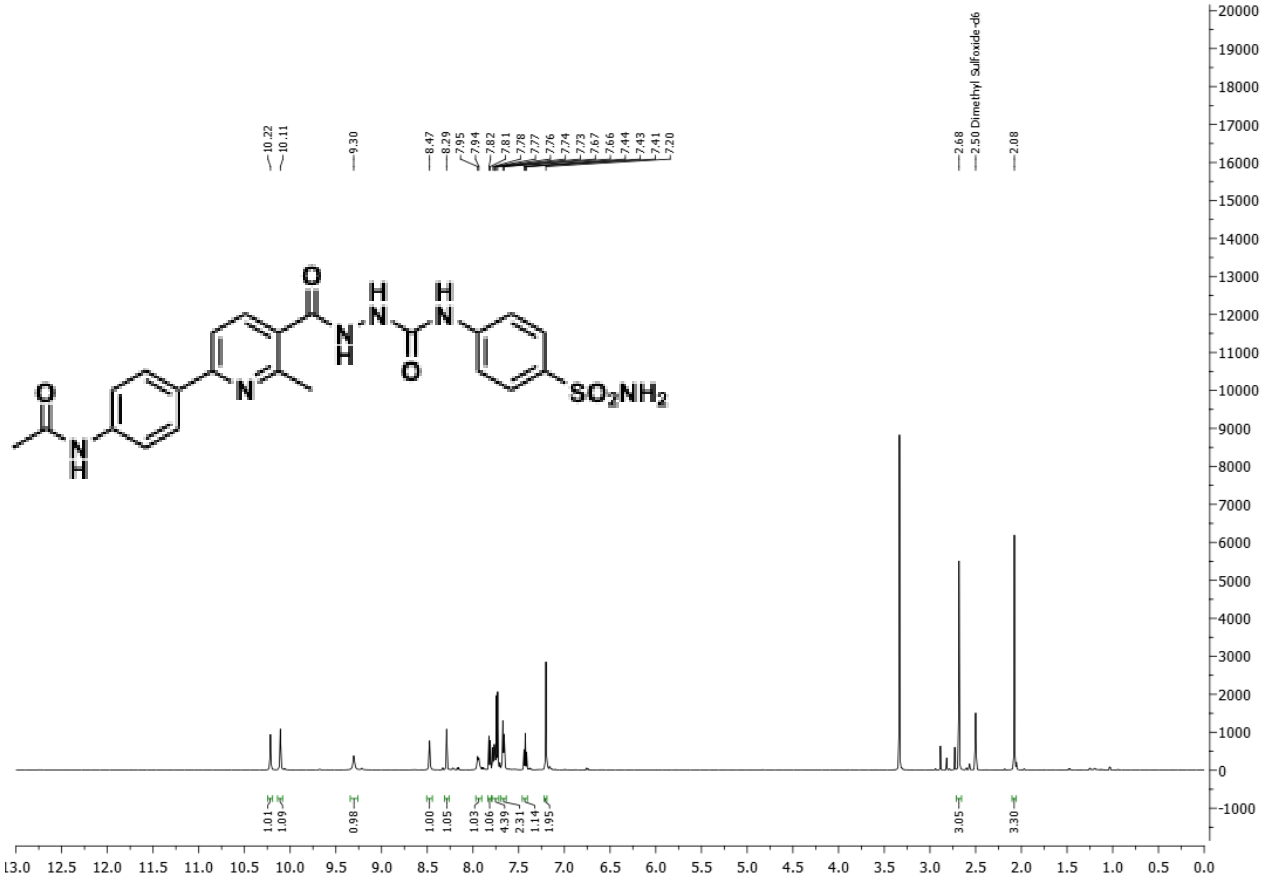

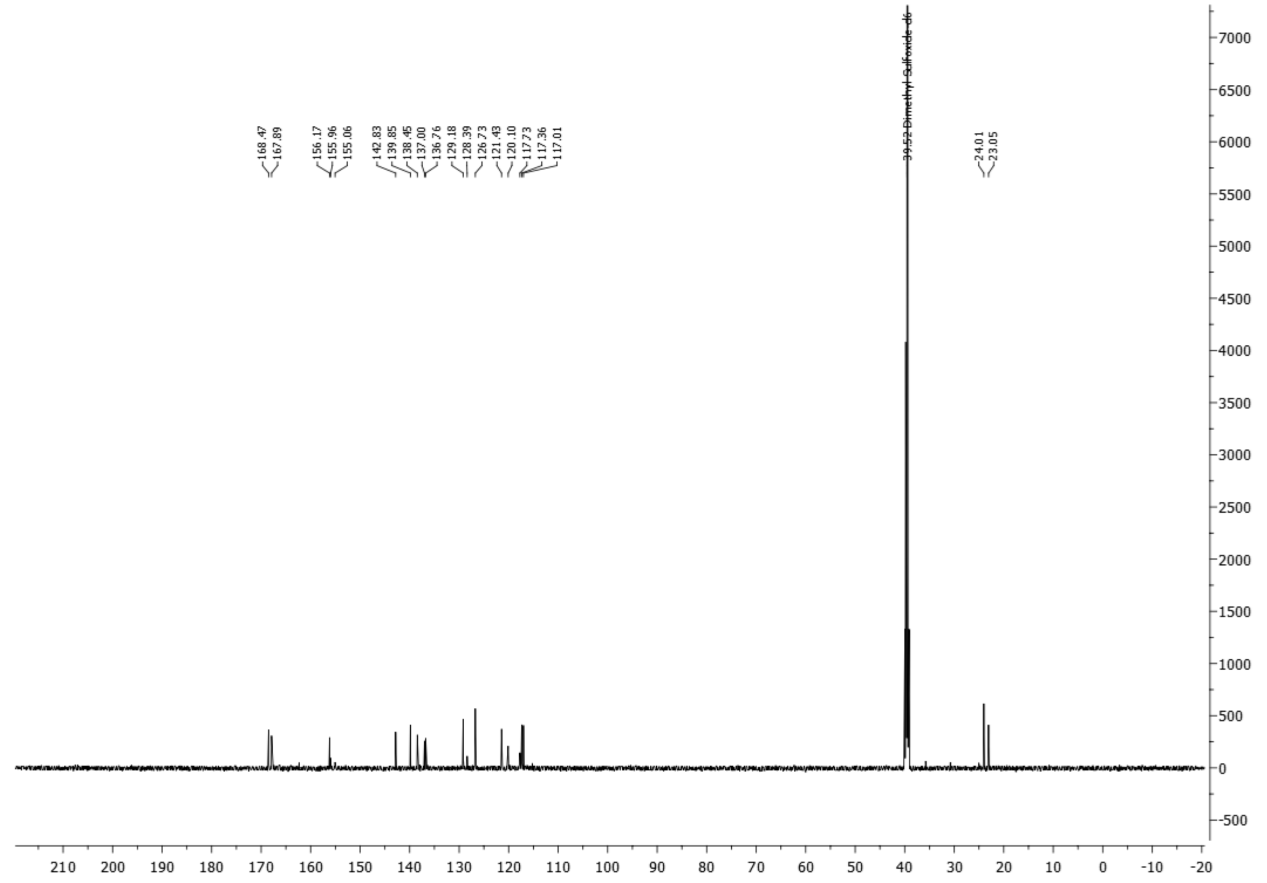

2-(6-(3,5-bis(trifluoromethyl)phenyl)-2-methylnicotinoyl)-N-(4-sulfamoylphenyl)hydrazinecarboxamide ***5a***

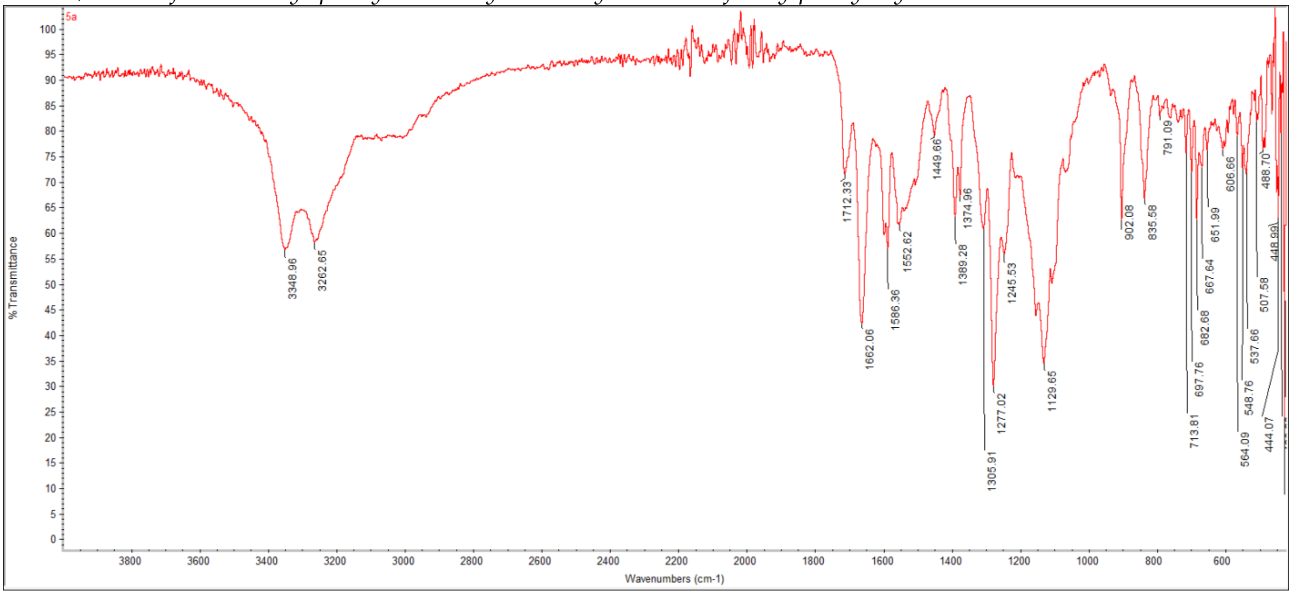

2-(6-(benzofuran-2-yl)-2-methylnicotinoyl)-N-(4-sulfamoylphenyl)hydrazinecarboxamide ***5b***

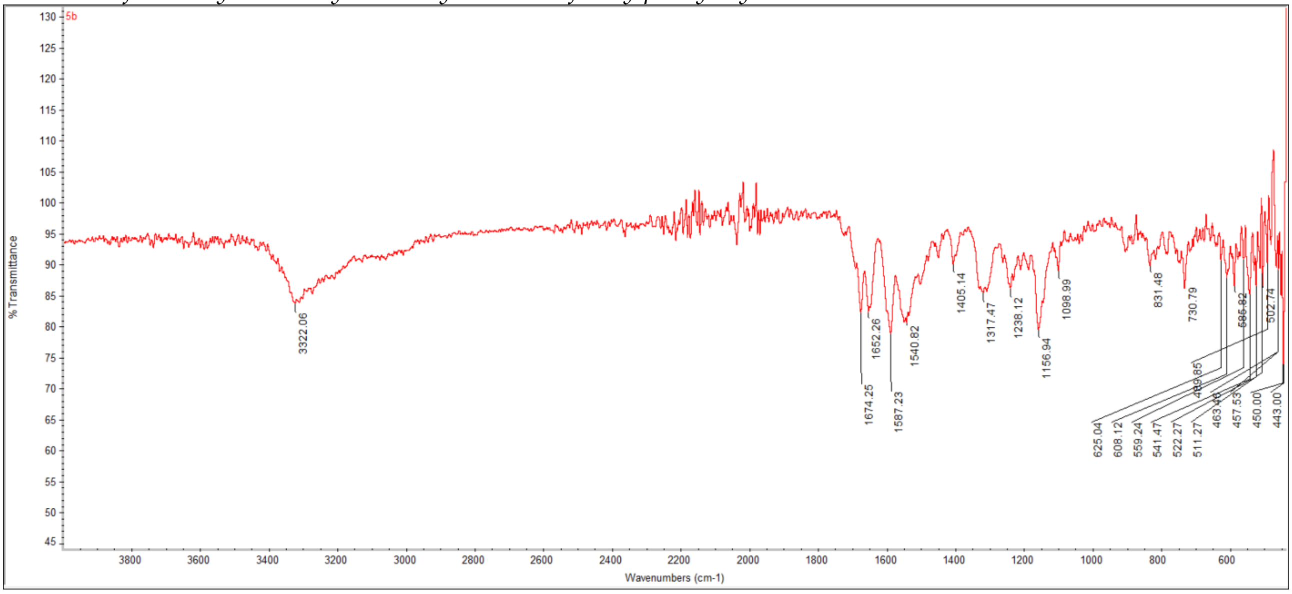

2-(2-methyl-6-(m-tolyl)nicotinoyl)-N-(4-sulfamoylphenyl)hydrazinecarboxamide ***5c***

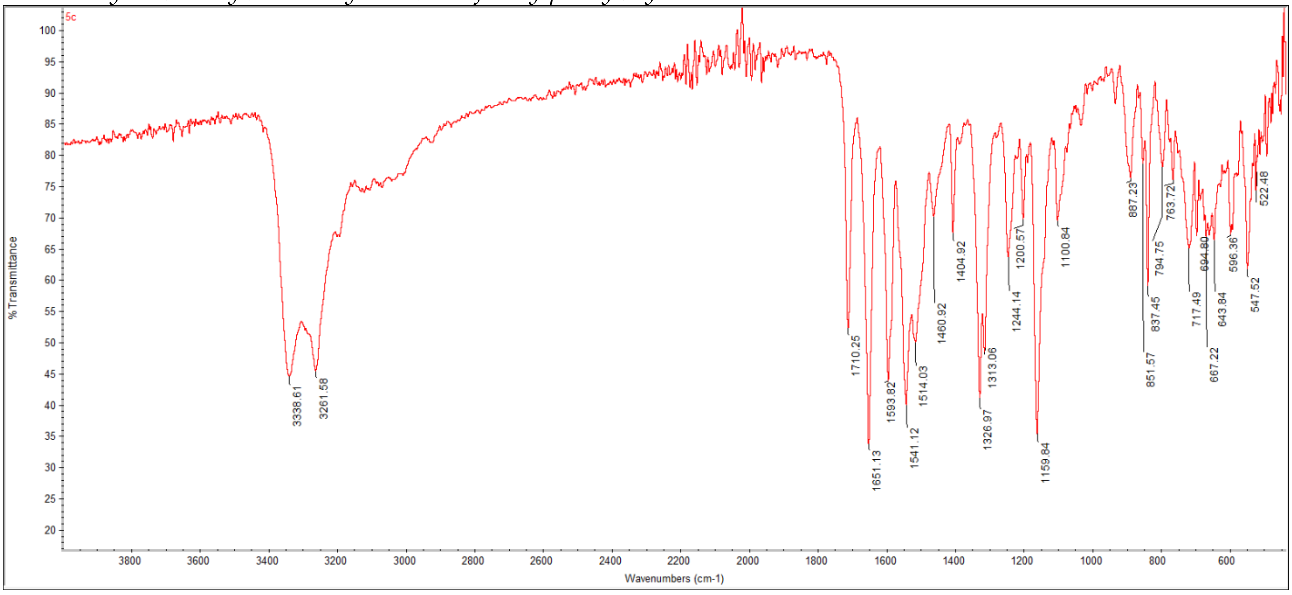

2-(6-(3-bromophenyl)-2-methylnicotinoyl)-N-(4-sulfamoylphenyl)hydrazinecarboxamide ***5d***

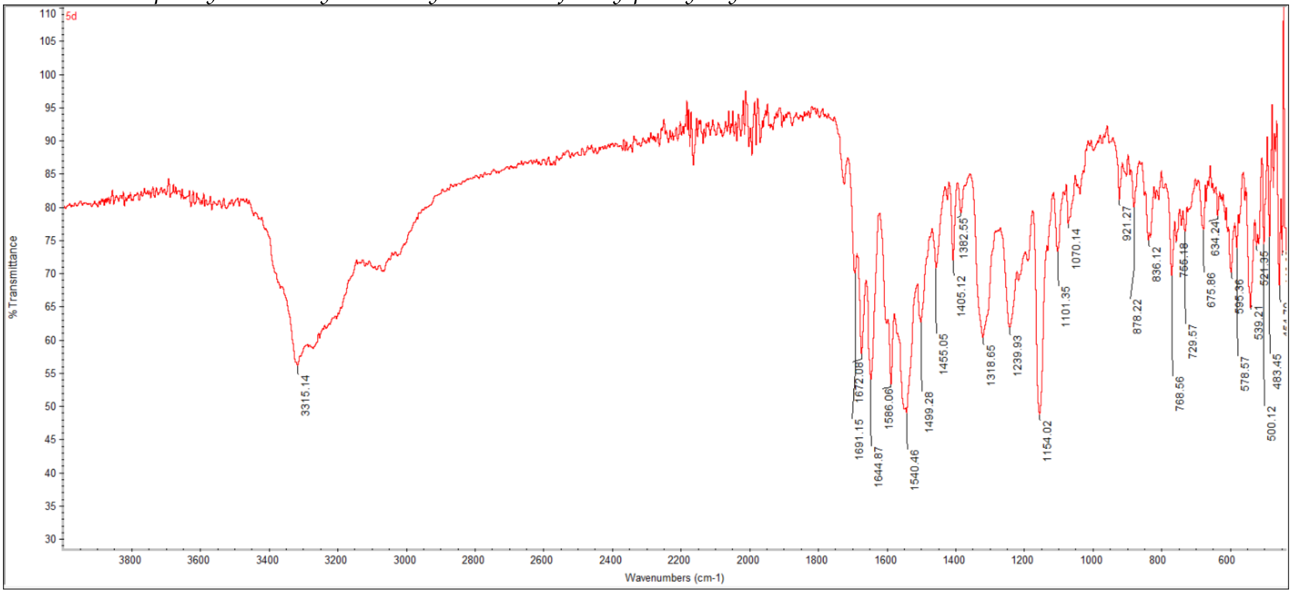

2-(6-(4-fluorophenyl)-2-methylnicotinoyl)-N-(4-sulfamoylphenyl)hydrazinecarboxamide ***5e***

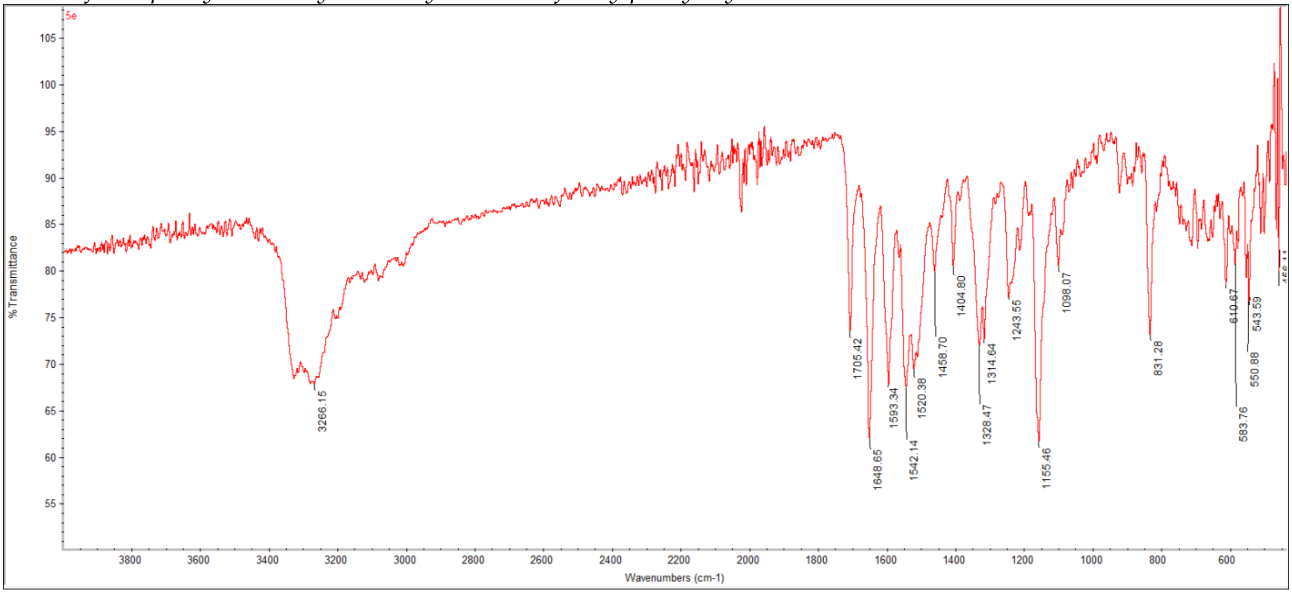

2-(2-methyl-6-(4-(trifluoromethyl)phenyl)nicotinoyl)-N-(4-sulfamoylphenyl)hydrazinecarboxamide ***5f***

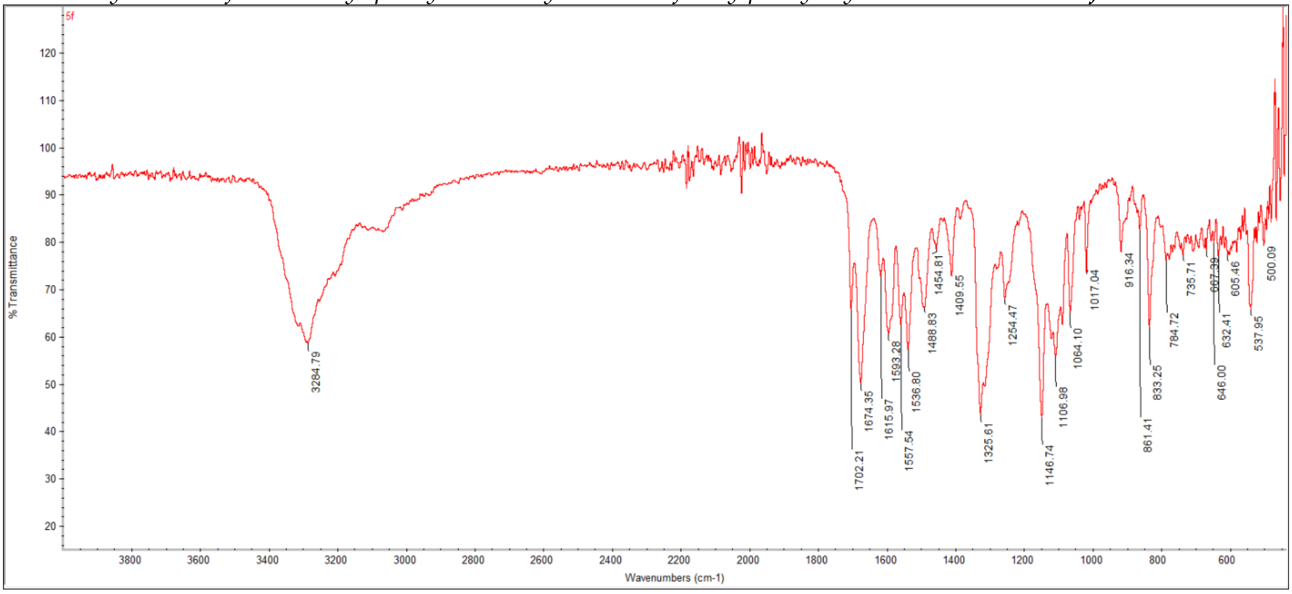

2-(2-methyl-6-(3,4,5-trimethoxyphenyl)nicotinoyl)-N-(4-sulfamoylphenyl)hydrazinecarboxamide ***5g***

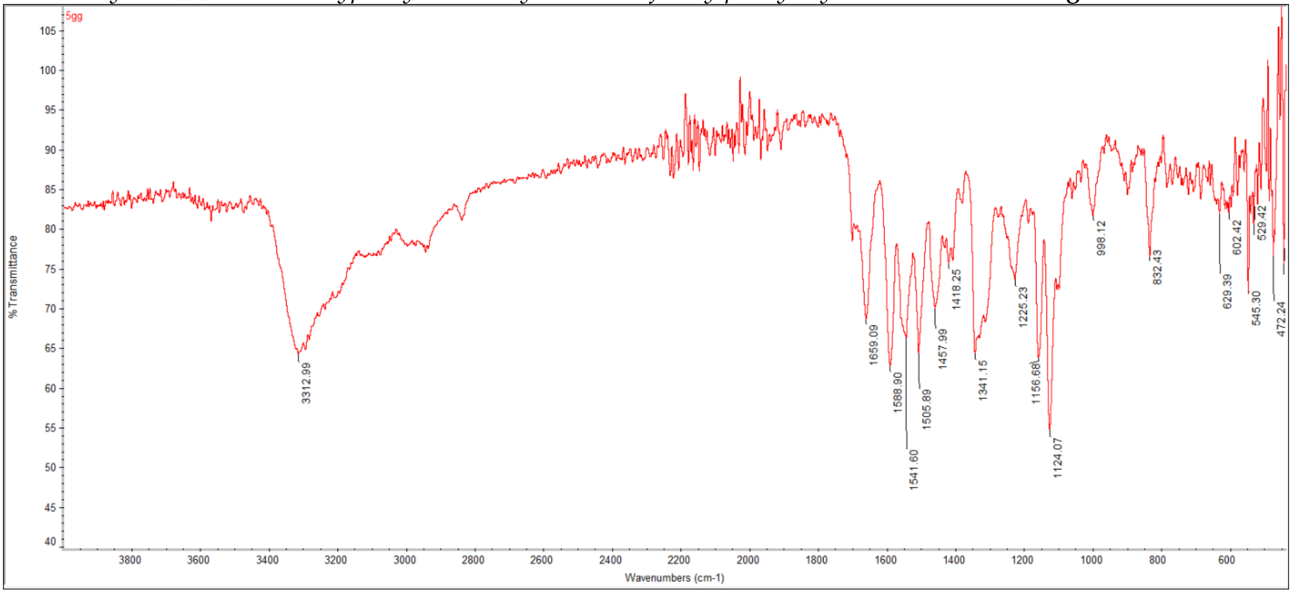

2-(6-(3,4-dimethoxyphenyl)-2-methylnicotinoyl)-N-(4-sulfamoylphenyl)hydrazinecarboxamide ***5h***

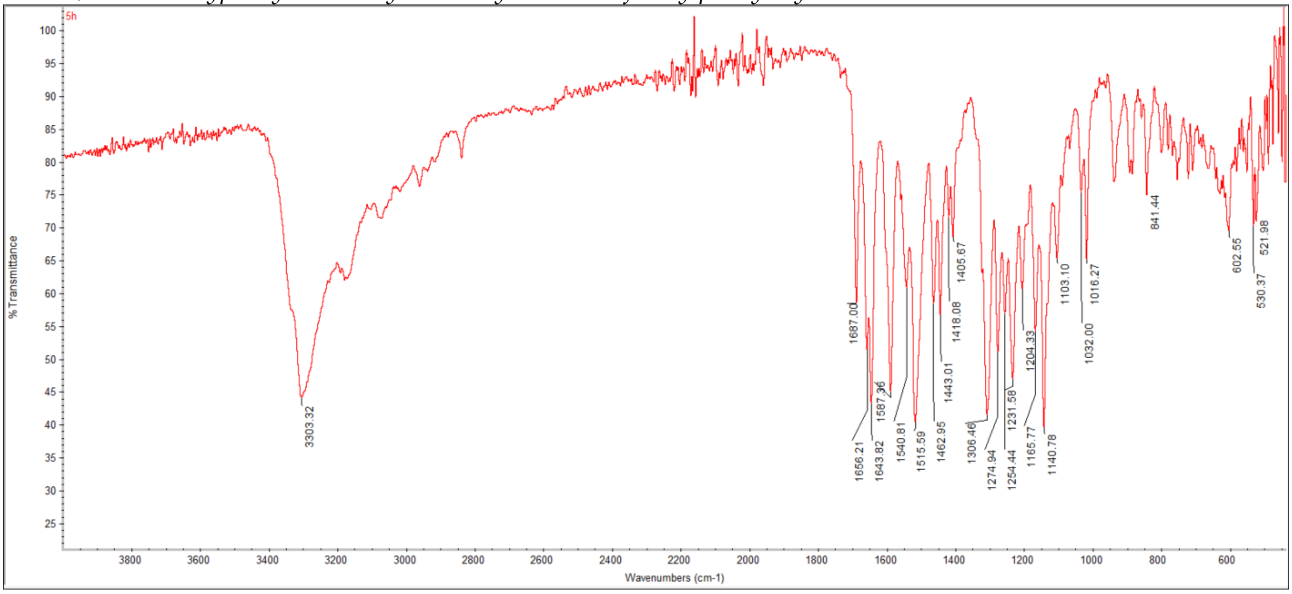

2-(2-methyl-6-(4-(methylsulfonamido)phenyl)nicotinoyl)-N-(4-sulfamoylphenyl)hydrazinecarboxamide ***5i***

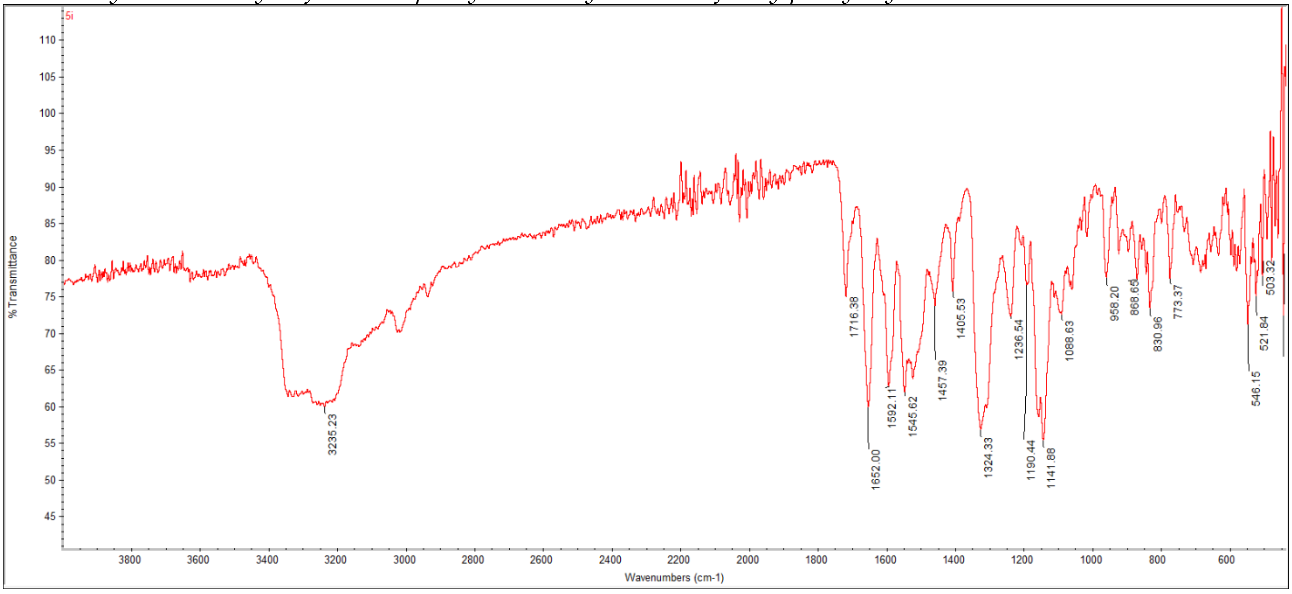

2-(6-(4-acetamidophenyl)-2-methylnicotinoyl)-N-(4-sulfamoylphenyl)hydrazinecarboxamide ***5j***

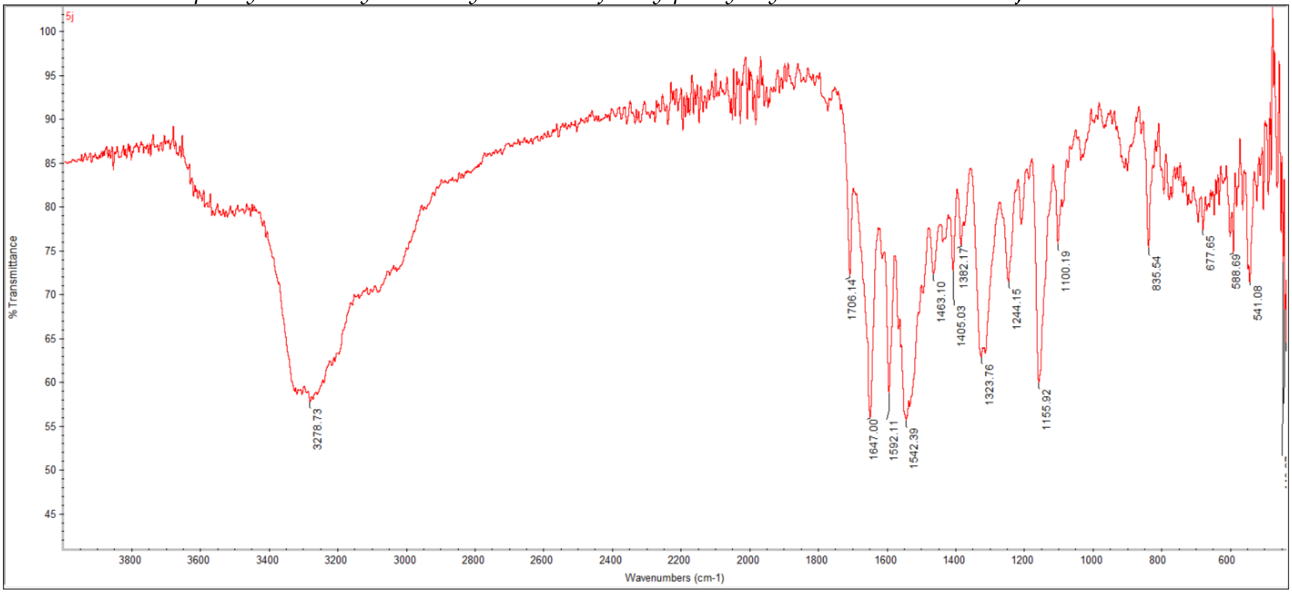

Supplement: Supplementary file 1 [file pharmaceuticals-19-00290-s001.zip › pharmaceuticals-4125586-supplementary.pdf]
